# How different are objective operationalizations of walkability for older adults compared to the general population? A systematic review

**DOI:** 10.1186/s12877-022-03233-x

**Published:** 2022-08-15

**Authors:** Zeynep S. Akinci, Xavier Delclòs-Alió, Guillem Vich, Deborah Salvo, Jesús Ibarluzea, Carme Miralles-Guasch

**Affiliations:** 1 Grup d’Estudis en Mobilitat, Transport i Territori (GEMOTT), Departament de Geografia, Universitat Autònoma de Barcelona, Edifici B, Campus de Bellaterra, 08193 Cerdanyola del Vallès, Barcelona Spain; 2 Grup de Recerca en Anàlisi Territorial i Estudis Turístics (GRATET), Departament de Geografia, Universitat Rovira i Virgili, Vila-seca, Spain; 3 ISGlobal (Barcelona Institute for Global Health), Doctor Aiguader, 88, 08003 Barcelona, Spain; 4 People, Health and Place Unit; Prevention Research Center in St. Louis; Brown School; Washington University in St Louis , St. Louis, Missouri, USA; 5 Ministry of Health of the Basque Government, Sub-Directorate for Public Health and Addictions of Gipuzkoa, 20013 San Sebastian, Spain; 6 Faculty of Psychology of the University of the Basque Country, 20018 San Sebastian, Spain; 7 Spanish Consortium for Research on Epidemiology and Public Health (CIBERESP), 28029 Madrid, Spain; 8 Biodonostia Health Research Institute, Environmental Epidemiology and Child Development Group, 20014 San Sebastian, Spain; 9 Institut de Ciència i Tecnologia Ambientals (ICTA), Universitat Autònoma de Barcelona – Edifici ICTA-ICP, Campus de Bellaterra, 08193 Cerdanyola del Vallès, Barcelona Spain

**Keywords:** Walkability, Older adults, Walking, Physical activity, Systematic literature review, Built environment

## Abstract

**Background:**

Walking is an essential activity for everyone and for older adults in particular, given that it is the most accessible form of physical activity and one of the healthiest transportation modes. Understanding how walkability (the potential of the environment to enable and/or encourage walking) has been objectively measured and analyzed for older adults is critical to create more inclusive, healthy, and sustainable environments and to promote healthy aging. Despite the numerous reviews on physical activity among older adults and its relationship with the built environment, the literature still lacks comparison reviews focusing specifically on objective operationalizations of walkability for older adults vs. the general population.

**Methods:**

We conducted a systematic review of 146 empirical studies that measured walkability objectively in relation to walking-related outcomes. We compared studies focused on older adults (*n* = 24) and the general population (*n* = 122). Content analysis included the characteristics of the study design, walkability measures, spatial extent, and associations found between walkability and walking-related outcomes.

**Results:**

In both groups of publications, the majority of studies were conducted in the US, Canada, and Europe, and largely in high-income countries. They were mostly published in health-related journals and used cross-sectional designs, operationalized walkability by using indexes, employed self-reported measures for walking-related outcomes, and found positive associations between walkability and walking outcomes. However, we observed some differences among studies focusing on older adults. Compared to studies focusing on the general population, a larger proportion of studies on older adults was conducted in the Middle East and Asia, and they used longitudinal designs, mixed methods to measure walking-related outcomes, variables related with land-use characteristics, safety from traffic and crime, and greenery, and a larger proportion found positive, as well as no associations between walkability and walking-related outcomes.

**Conclusion:**

Although there is a promising increase in interest in older adults-focused walkability studies in the last decade, there is still a need for more studies focusing on different settings, using wider spatial extents, longitudinal designs, objective or mixed methods to collect outcome data, and specific variables and/or specially created indexes for older adults and for settings.

**Supplementary Information:**

The online version contains supplementary material available at 10.1186/s12877-022-03233-x.

## Introduction

Walking is one of the most accessible, economically viable, democratic, communal, sustainable, environmentally friendly, and healthiest forms of transportation [1–4]. It is also the easiest way of including physical activity (PA) into daily life routines while helping to achieve recommendations for a physically and mentally healthy life (i.e., 150–300 minutes/ week of moderate-intensity activity for adults aged 18–64 years, and a minimum of 150 minutes/ week for persons ≥65 years) [5, 6]. Additionally, for specific population groups, such as older adults (≥65 years), walking is the most common, if not the only, type of PA [7]. Yet, engaging in this activity is related to various factors.

Among many other factors, walking depends on who is undertaking this activity (i.e., the characteristics of individuals). Some population groups, for instance, older adults, are less involved in this activity due to factors such as increased physical limitations compared to other age groups. Walking also depends on where it takes place, since the characteristics of an environment could encourage or limit this activity. Some environmental features, such as dimly lit streets, steps, steep hills, or broken pavements might become a barrier for walking among some groups such as older adults more than others [8], due to the decrease in the level of “individual competence” [9] to cope with the “environmental press” ([9], pp.25, [10], pp385–396). Thus, some environments could be more “walking-friendly” or walkable than others, for different types of individuals.

The definition of walkability varies vastly in the literature, and depends on “who is asking” [11] or personal perspective. The most common definition has been the walking/ pedestrian friendliness of a given place [12]. However, more detailed definitions such as how traversable, compact, safe, lively and sociable, physically enticing, or exercise-inducing an environment is, have also been used [13]. Walkability has also been defined as a complex and multidimensional concept, whose dimensions are measurable “individually or combined into an index” [13]. Studies measuring walkability of a place have received greater scholarly attention in the last decades in different countries, under the scope of various research fields, and using a wide array of variables and operationalization methods [14]. Many studies have associated walkability with PA outcomes, and while the results generally show a positive association between the two, variations for different pedestrian groups, such as children, adults, older adults, or impaired pedestrians, are also highlighted [12, 15]. Some studies have employed subjective measures (e.g., perceptions), while others have preferred measuring walkability objectively (e.g., by using Geographic Information Systems - GIS). Although some studies on adults presented partial agreement between subjective and objective measures of walkability [16], high misperception levels were also highlighted in other studies [17]. Studies on older adults that used objective measures in their analysis generally presented stronger associations [12, 18].

Various reviews on walkability studies have to date focused on how differently walkability is defined in the literature [11, 13], how it is operationalized, and how it could contribute to more PA engagement [19–23], or on the trends that walkability research has followed throughout the years [14]. Some reviews narrowed down their scope to specific groups such as adults [24] or children [15]. Despite the high importance of walking among older adults and theirs being the most sedentary group (with about 60 to 80% of their daily time spent physically inactive), to the best of our knowledge, none of the systematic reviews on the PA of older adults [25–29] focused specifically on walkability, but rather included various built environment characteristics in their studies. Only one systematic review focused on the relationship between walkability and the PA of older adults [30]; however, their specific aim was to examine the impacts of stairs on this relationship. Thus, we believe that there is a need for a systematic review which summarizes how objective walkability has been operationalized to date, its relationship with walking outcomes, and how these differed for older adults, for whom walking is particularly essential. By detecting the gaps in the literature, and summarizing the methodologies of previous studies, this review could help to inform future literature reviews and empirical analyses that share similar aims. Additionally, by highlighting how objective walkability measures differ for older adults compared to the general population, this review could also offer insight for urban designers, planners, and/or local governments.

Following this introduction, the next section provides a description of the methodology employed for this systematic literature review. Then in the results section, we first present the pattern of demographic groups included in all reviewed publications, and then we compare the descriptive results from papers focusing only on older adults and those exploring the general population. Finally, we discuss these results, and end the paper with concluding remarks.

## Methods

### Search strategy

**Fig. 1:**
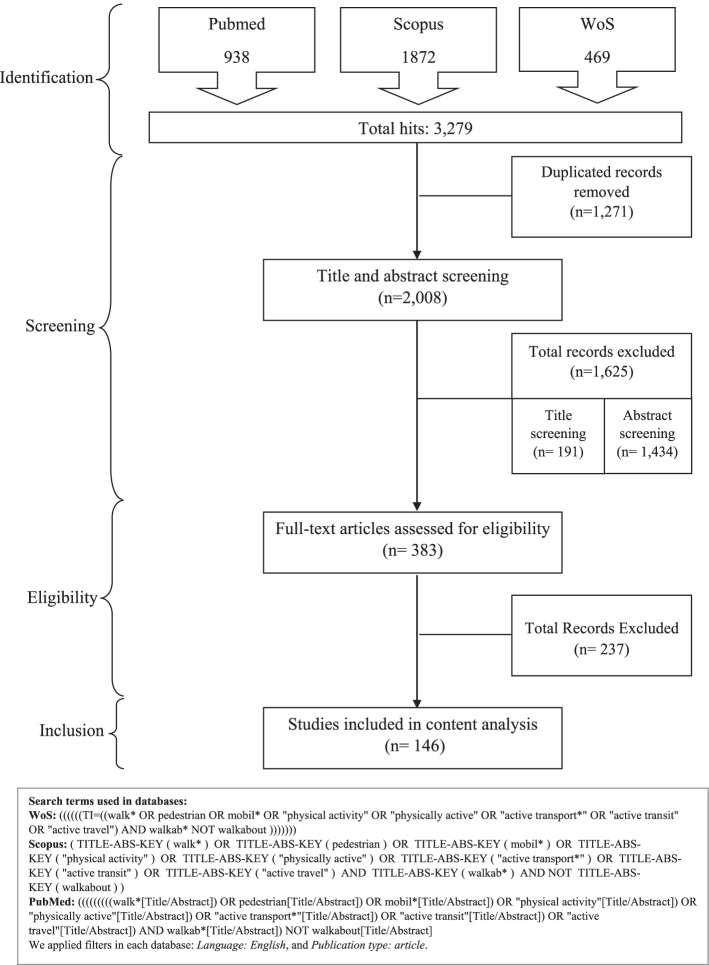
PRISMA flow chart for systematic literature search and search terms used in the three databases

This systematic review followed the PRISMA (Preferred Reporting Items of Systematic Review and Meta-Analysis) guidelines [31]. First, we defined a query logic based on keywords related to walkability and walking-related PA. Second, we ran initial tests in different databases, and conducted the final search on June 25, 2019, in three electronic databases: PubMed, Scopus, and Web of Science (WoS) (Fig. 1). In order to include the seminal publications meeting our criteria, we did not set a start date for the search.

### Inclusion and exclusion criteria

Papers were included only if they were, 1) focusing on measuring walkability (i.e., only those with an explicit mention of walkability in their titles or abstracts, methods, and results sections, excluding those using walkability only for sample recruitment, for instance); 2) measuring walkability objectively (with GIS, or environmental/ street audits conducted by trained people); 3) having subjectively or objectively measured (e.g., by using self-reports or accelerometers) walking-related outcomes (excluding those combining different types of PA, such as cycling, gardening, skating, etc., under one category such as total PA or active commuting); 4) relating these walking-related outcomes with walkability; 5) original empirical research published in peer-reviewed journals; and 6) written in English.

### Study selection

Study selection was conducted in four phases for the first part of the review (Fig. 1). After removing the duplicates from the total records retrieved from the database search (*n* = 3279), we first included 2008 manuscripts in the phase of title screening and then abstract screening for relevance. According to the selection criteria, a total of 1625 papers were excluded at these phases. Then full texts of the remaining papers (*n* = 383) were reviewed. Given the detailed information gathered at this stage, a further 237 papers were excluded. Finally, the remaining 146 papers were included in the content analysis. For consistency, all phases were completed by the first author (ZSA). After each phase, the second and third authors (XDA and GV, respectively) individually screened a random selection of 20% of the publications to eliminate the risk of bias and confirm the correctness of the selection. In case of doubt or disagreement, discussions of the papers among the authors took place until a joint decision was made.

### Data extraction and content analysis

For all included publications (*n* = 146) data were extracted and assessed under five main categories. The reasons and details of the categorization and coding used in these categories are explained briefly below, and in detail in Supplementary Material.

1. General study characteristics: Publication year, Journal field, Geographical context (study setting), Demographic group under study.
2. Characteristics of the study design: Research design (cross-sectional, longitudinal, or mixed), Spatial data collection method (GIS or audit), Outcome data collection method (objective, subjective, or mixed methods).
3. Characteristics of walkability measures: Operationalization of walkability (indexes or separate variables), Walkability variables used.
4. Spatial extent and unit: Spatial extent (residential area, school site, etc.), Spatial unit (administrative units, statistical units, buffers, etc.), Buffer type (circular, street network, or sausage buffer), and buffer size.
5. Associations found between walkability and walking-related outcomes (coded as *positive*, *negative*, *no association*, *mixed* -for studies providing results for different population groups or settings- or *partial* -for studies providing results for different buffer sizes or different walking-related outcomes and/or, studies providing different associations for each walkability variable used, and when this difference is not acute, e.g., two no associations, two positive, and three negative associations).

After analyzing the contents of all publications meeting our criteria (*n* = 146) according to the abovementioned fields, we stratified the analysis to compare publications focusing only on older adults (*n* = 24), and the general population (*n* = 122).

## Results

Among all publications included in the content analysis (*n* = 146), 50.7% (*n* = 74) focused on adults, although the definition of this group varied vastly across studies (See Supplementary Material, Section 1.1.4 for further information). 17.8% (*n* = 26) focused on “all population” in their analysis while 15.1% focused on young people (*n* = 22). Finally, publications focusing on older adults formed 16.4% of the analyzed studies, with 24 publications.

The results of the content analysis are presented in Table 1, Table 2, and Table 3. In addition, Table 4 presents detailed list of publications in relation to all variables included in the content analysis.

**Table 1:** Proportion of different variables included in the analysis among papers focusing on older adults vs. the general population

General study characteristics	Older adults *n* = 24 (100%)	General population *n* = 122 (100%)
**Publication year**		
2016–2019	11 (45.8)	57 (46.7)
2011–2015	10 (41.7)	53 (43.4)
2005–2010	3 (12.5)	12 (9.8)
**Journal field**		
Health	13 (54.2)	77 (63.1)
Inter- or multi-disciplinary	9 (37.5)	38 (31.1)
Transportation or urban studies	1 (4.2)	4 (3.3)
Environment or geography	1 (4.2)	3 (2.5)
**Geographical context**		
US and Canada	12 (50.0)	68 (55.7)
Europe	7 (29.2)	25 (20.5)
Middle East and Asia	4 (16.7)	4 (3.3)
Oceania	1 (4.2)	21 (17.2)
Latin America	0 (0.0)	2 (1.6)
Multiple country	0 (0.0)	2 (1.6)
**Characteristics of the study design**		
**Research design**		
Cross-sectional	18 (75.0)	105 (86.1)
Longitudinal	5 (20.8)	16 (13.1)
Mixed	1 (4.2)	1 (0.8)
**Spatial data collection method**		
GIS	22 (91.7)	114 (93.4)
Audits	2 (8.3)	8 (6.6)
**Outcome data collection method**		
Self-reported	14 (58.3)	70 (57.4)
Device	3 (12.5)	31 (25.4)
Mixed	7 (29.2)	21 (17.2)
**Characteristics of the walkability measures**		
**Operationalization of walkability**		
Index	19 (79.2)	112 (91.8)
Separate variables	5 (20.8)	10 (8.2)
**Spatial extent and unit**		
**Spatial extent**		
Residential	24 (100.0)	107 (87.7)
School site	0 (0.0)	5 (4.1)
Residential + Workplace	0 (0.0)	4 (3.3)
Residential + School site	0 (0.0)	3 (2.5)
Other	0 (0.0)	3 (2.5)
**Spatial unit**		
Buffer	17 (70.8)	82 (67.2)
Statistical units	4 (16.7)	25 (20.5)
Administrative units	3 (12.5)	9 (7.4)
Combination	0 (0.0)	3 (2.5)
Other	0 (0.0)	3 (2.5)
**Associations found between walkability and walking-related outcomes**		
Positive	15 (62.5)	74 (60.7)
No association	5 (20.8)	18 (14.8)
Partial	3 (12.5)	20 (16.4)
Mixed	1 (4.2)	5 (4.1)
Negative	0 (0.0)	5 (4.1)

**Table 2:** Proportion of walkability variables used among papers focusing on older adults vs. the general population

	Older adults (*n* = 167 variables used in 24 studies) n (%)	General population (*n* = 518 variables used in 122 studies) n (%)
Land use characteristics	56 (33.5)	85 (16.4)
Safety from traffic	20 (12)	26 (5)
Street connectivity	19 (11.4)	116 (22.4)
Street design	19 (11.4)	55 (10.6)
Activity and destination density and access to services	18 (10.8)	92 (17.8)
Population density	15 (9)	86 (16.6)
Safety from crime	8 (4.8)	14 (2.7)
Greenery	8 (4.8)	20 (3.9)
Transportation accessibility	3 (1.8)	16 (3.1)
Topographic characteristics	1 (0.6)	6 (1.2)
Socioeconomic characteristics	0 (0)	2 (0.4)

**Table 3:** Proportion of buffer types and sizes used among studies focusing on older adults vs. general population

Buffer type	Older adults (***n*** = 17 buffer types used in 17 studies using buffers) n (%)	General population (***n*** = 91 buffer types used in 82 studies using buffers) n (%)
Street network buffer	9 (53)	47 (51.6)
Circular buffer	8 (47)	42 (46.2)
Sausage buffer	0 (0)	2 (2.2)
**Buffer size**	**Older adults (*n*** **= 20 buffer sizes used in 17 studies using buffers) n (%)**	**General population (*n*** **= 125 buffer sizes used in 82 studies using buffers) n (%)**
50 m	0 (0)	1 (1)
200 m	1 (5)	1 (1)
250 m	0 (0)	1 (1)
400 m	3 (15)	9 (7)
500 m	3 (15)	9 (7)
600 m	0 (0)	1 (1)
800 m	1 (5)	11 (9)
1000 m	5 (25)	31 (25)
1200 m	0 (0)	1 (1)
1500 m	0 (0)	2 (2)
1600 m	0 (0)	14 (11)
1700 m	0 (0)	1 (1)
2000 m	1 (5)	4 (3)
2500 m	1 (5)	1 (1)
3000 m	5 (25)	37 (30)
4830 m	0 (0)	1 (1)
**Buffer size ≤ 1000 m or > 1000 m n (%)**		
≤ 1000 m	13 (65)	64 (51)
> 1000 m	7 (35)	61 (49)

**Table 4:** Content analysis of the reviewed publications

Demographic group under study	# of articles	Reference	
**All population**	26	[32–57]	
**Adults**	74	[58–131]	
**Young people**	22	[132–153]	
**Older adults**	24	[154–177]	
**Publication period**	**# of articles**	**Reference**	
		**Older adults**	**General population**
**2005–2010**	Older adults: 3 General population: 12	[157, 165, 170]	[58, 59, 61, 63, 67, 86, 100, 107, 124, 126, 135, 137]
**2011–2015**	Older adults: 10 General population: 53	[158–160, 162, 167, 171, 172, 174–176]	[32, 34–37, 41, 44, 45, 47, 48, 51, 54–56, 60, 64, 66, 68, 74, 75, 77, 80, 81, 85, 87, 93, 95, 96, 98, 99, 103, 105, 108, 110–112, 114, 116, 117, 121, 123, 128, 130, 132, 134, 136, 138, 142, 144, 147–149, 152]
**2016–2019**	Older adults: 11 General population: 57	[154–156, 161, 163, 164, 166, 168, 169, 173, 177]	[33, 38–40, 42, 43, 46, 49, 50, 52, 53, 57, 62, 65, 69–73, 76, 78, 79, 82–84, 88–92, 94, 97, 101, 102, 104, 106, 109, 113, 115, 118–120, 122, 125, 127, 129, 131, 133, 139–141, 143, 145, 146, 150, 151, 153]
**Journal field**	**# of articles**	**Reference**	
		**Older adults**	**General population**
**Health**	Older adults: 13 General population: 77	[155, 157, 159, 160, 162–165, 170–172, 174, 175]	[32, 34, 35, 37, 39, 40, 42, 43, 46, 50, 54, 56, 58, 59, 65, 66, 68, 69, 71, 73–77, 80–82, 84, 85, 87, 88, 90–92, 96–101, 103, 104, 106, 108–113, 115, 116, 118–122, 124, 125, 128, 129, 132, 133, 135–141, 143, 144, 146, 148, 150–153]
**Inter- or multi-disciplinary**	Older adults: 9 General population: 38	[154, 158, 161, 166–169, 173, 177]	[33, 41, 44, 47–49, 51, 52, 55, 57, 62, 63, 67, 70, 72, 78, 79, 83, 86, 89, 93–95, 102, 105, 107, 114, 117, 123, 126, 127, 130, 131, 134, 142, 145, 147, 149]
**Transportation or urban studies**	Older adults: 1 General population: 4	[176]	[45, 53, 61, 64]
**Environment or geography**	Older adults:1 General population: 3	[156]	[36, 38, 60]
**Geographical context**	**# of articles**	**Reference**	
		**Older adults**	**General population**
**US and Canada**			
*US*	Older adults: 9 General population: 46	[155, 157, 162, 165, 167, 170, 171, 174, 175]	[32, 38, 42, 45, 49, 50, 55, 58–61, 65, 67, 68, 73–75, 77–79, 81–87, 89, 90, 98, 99, 103, 104, 106, 107, 118–122, 135, 136, 143, 145, 146, 150]
*Canada*	Older adults: 3 General population: 22	[160, 163, 164]	[35, 40, 46, 48, 53, 54, 66, 70–72, 76, 80, 94, 95, 101, 105, 109, 125, 127, 134, 152, 153]
**Europe**			
*Austria*	General population: 1	–	[69]
*Belgium*	Older adults: 3 General population: 7	[158, 159, 177]	[123, 124, 126, 133, 144, 147, 148]
*Czech Republic*	General population: 1	–	[63]
*Denmark*	General population: 1	–	[114]
*Finland*	Older adults: 1	[173]	–
*France*	General population: 2	–	[62, 97]
*Germany*	Older adults: 1 General population: 2	[154]	[41, 132]
*Ireland*	General population: 1	–	[131]
*Portugal*	General population: 1	–	[102]
*Spain*	Older adults: 1 General population: 2	[169]	[140, 141]
*Sweden*	General population: 4	–	[34, 44, 51, 117]
*United Kingdom*	Older adults: 1 General population: 3	[161]	[57, 92, 113]
**Middle East and Asia**			
*Iran*	Older adults: 1	[176]	–
*Japan*	Older adults: 1 General population: 1	[166]	[39]
*Malaysia*	General population: 1	–	[129]
*Singapore*	Older adults: 1	[172]	–
*South Korea*	General population: 2	–	[37, 64]
*Taiwan*	Older adults: 1	[168]	–
**Oceania**			
*Australia*	Older adults: 1 General population: 16	[156]	[36, 47, 52, 56, 88, 91, 93, 96, 100, 110, 111, 115, 116, 130, 138, 149]
*New Zealand*	General population: 5	–	[128, 137, 139, 142, 151]
**Latin America**			
*Brazil*	General population: 1	–	[112]
*Mexico*	General population: 1	–	[108]
**Multiple country**	General population: 2	–	[33, 43]
**Reference**			
**Research design**	**# of articles**	**Older adults**	**General population**
**Cross-sectional**	Older adults: 18 General population: 105	[154–156, 158–165, 168, 169, 172, 174–177]	[33–35, 37–39, 41–47, 49–56, 58–71, 73, 75–85, 88, 90, 91, 93, 95–105, 107, 108, 110–121, 123–126, 128–144, 146–153]
**Longitudinal**	Older adults: 5 General population: 16	[157, 166, 167, 170, 171]	[32, 36, 40, 48, 57, 72, 74, 86, 87, 89, 94, 106, 109, 122, 127, 145]
**Mixed**	Older adults: 1 General population: 1	[173]	[92]
**Spatial data collection method**	**# of articles**	**Reference**	
		**Older adults**	**General population**
**GIS**	Older adults: 22 General population: 114	[154, 155, 157–173, 175–177]	[32–71, 73–76, 78–82, 84–103, 105–127, 129–138, 140, 141, 143–145, 147–153]
**Audits**	Older adults: 2 General population: 8	[156, 174]	[72, 77, 83, 104, 128, 139, 142, 146]
**Outcome data collection method**	**# of articles**	**Reference**	
		**Older adults**	**General population**
**Self-reported**	Older adults: 14 General population: 70	[154, 156, 157, 160, 164–166, 168–172, 175, 176]	[35, 37, 39–41, 45, 47–50, 52–58, 60, 61, 64–67, 69, 70, 75–77, 80, 81, 84–88, 91, 93–100, 102, 103, 105, 109–112, 114–116, 119–121, 123, 125, 127, 128, 130, 131, 134, 141, 145, 146, 149, 150, 152]
**Mixed**	Older adults: 7 General population: 21	[155, 158, 159, 163, 167, 173, 177]	[34, 36, 51, 59, 62, 68, 89, 90, 106, 107, 113, 117, 124, 126, 133, 137, 140, 142–144, 153]
**Device**	Older adults: 3 General population: 31	[161, 162, 174]	[32, 33, 38, 42–44, 46, 63, 71–74, 78, 79, 82, 83, 92, 101, 104, 108, 118, 122, 129, 132, 135, 136, 138, 139, 147, 148, 151]
**Operationalization of walkability**	**# of articles**	**Reference**	
		**Older adults**	**General population**
**Index**	Older adults: 19 General population: 112	[154, 157–160, 162–170, 172, 173, 175–177]	[33–36, 38–51, 53, 54, 56–59, 61–67, 69–77, 79, 81, 82, 84–135, 137–153]
**Separate variables**	Older adults: 5 General population: 10	[155, 156, 161, 171, 174]	[32, 37, 52, 55, 60, 68, 78, 80, 83, 136]
**Spatial domain**	**# of articles**	**Reference**	
		**Older adults**	**General population**
**Residential**	Older adults: 24 General population: 107	[154–177]	[32, 34–37, 40–59, 62–89, 91, 92, 94–133, 135, 138–140, 143–148, 151, 153]
**School site**	General population: 5	–	[134, 137, 149, 150, 152]
**Residential + Workplace**	General population: 4	–	[39, 61, 90, 93]
**Residential + School site**	General population: 3	–	[136, 141, 142]
**Other** (whole city, routes to parks, daily walking itineraries)	General population: 3	–	[33, 38, 60]
**Spatial extent**	**# of articles**	**Reference**	
		**Older adults**	**General population**
**Buffer**	Older adults: 17 General population: 82	[154–157, 160–166, 168, 170, 172–175]	[32–35, 37, 39–44, 46–54, 56, 57, 59, 62, 63, 65–69, 71–76, 78–81, 83, 84, 86, 87, 89, 92, 94–98, 101–106, 108, 109, 113, 115, 116, 118–122, 127, 130–132, 134–136, 138, 142, 143, 145, 146, 149, 151, 152]
**Statistical units**	Older adults: 4 General population: 25	[158, 159, 171, 177]	[55, 58, 64, 82, 85, 88, 93, 100, 110–112, 114, 123–126, 128, 133, 137, 139–141, 144, 147, 148]
**Administrative units**	Older adults: 3 General population: 9	[167, 169, 176]	[45, 70, 77, 91, 99, 107, 117, 129, 153]
**Combination**	General population: 3	–	[36, 38, 90]
**Other** (street segments, country level, enrollment zones)	General population: 3	–	[60, 61, 150]
		**Reference**	
**Buffer type**	**# of articles**	**Older adults**	**General population**
**Street network buffer**	Older adults: 9 General population: 47	[154, 155, 160, 162–166, 174]	[32, 34, 36, 37, 39–44, 46, 47, 49, 50, 54, 56, 57, 59, 63, 66, 67, 69, 71, 75, 76, 79, 80, 83, 86, 87, 92, 95, 96, 98, 102–104, 108, 113, 116, 131, 132, 135, 138, 142, 143, 146]
**Circular buffer**	Older adults: 8 General population: 42	[156, 157, 161, 168, 170, 172, 173, 175]	[33, 35, 38, 41, 42, 48, 51, 53, 62, 65, 68–70, 72–75, 78, 79, 81, 84, 89, 90, 94, 97, 101, 105, 106, 109, 115, 118–122, 127, 130, 134, 136, 145, 149, 152]
**Sausage buffer**	General population: 2	–	[52, 151]
**Combination**	General population: 4	–	[41, 42, 69, 75] *References are also included in related categories above
**Buffer size**	**# of articles**	**Reference**	
		**Older adults**	**General population**
**50 m**	General population: 1	–	[118]
**200 m**	Older adults: 1 General population: 1	[174]	[47]
**250 m**	General population: 1	–	[151]
**400 m**	Older adults: 3 General population: 9	[154, 156, 170]	[47, 49, 52, 73, 83, 92, 102, 104, 135]
**500 m**	Older adults: 3 General population: 9	[162, 166, 172]	[35, 40, 43, 71, 72, 108, 131, 136, 151]
**600 m**	General population: 1	–	[49]
**800 m**	Older adults: 1 General population: 11	[170]	[47, 49, 52, 57, 78, 87, 90, 92, 102, 135, 142]
**1000 m**	Older adults: 5 General population: 31	[155, 157, 165, 166, 173]	[32, 34, 37, 39, 40, 42–44, 49, 51, 59, 63, 66–69, 79, 103, 106, 108, 113, 115, 116, 131, 132, 134, 142, 143, 146, 151, 152]
**1200 m**	General population: 1	–	[49]
**1500 m**	General population: 2	–	[49, 69]
**1600 m**	General population: 14	–	[41, 47, 49, 52, 56, 76, 80, 86, 87, 92, 95, 96, 98, 135]
**1700 m**	General population: 1	–	[49]
**2000 m**	Older adults: 1 General population: 4	[161]	[79, 138, 149, 151]
**2500 m**	Older adults: 5 General population: 37	[160, 163, 164, 168, 175]	[33, 35, 38, 41, 46, 48, 50, 53, 54, 62, 65, 68, 70, 72–76, 79, 81, 84, 89, 90, 94, 97, 101, 105, 106, 109, 115, 119–122, 127, 130, 145]
**3000 m**	Older adults: 1 General population: 1	[157]	[79]
**4830 m**	General population: 1	–	[87]
**Associations found between walkability and walking-related outcomes**	**# of articles**	**Reference**	
		**Older adults**	**General population**
**Positive**	Older adults: 15 General population: 74	[154, 155, 158, 160, 162, 164–167, 169, 171, 173, 174, 176, 177]	[33, 34, 36–38, 41–49, 51, 52, 55, 58–64, 66–70, 73–76, 78–82, 84, 85, 88, 91, 93–95, 97, 102, 106, 107, 112–117, 119, 120, 123–133, 135, 140–142, 146, 149]
**No association**	Older adults: 5 General population: 18	[156, 168, 170, 172, 175]	[35, 39, 40, 56, 57, 65, 71, 72, 83, 89, 99, 101, 105, 137, 138, 145, 150, 153]
**Partial**	Older adults: 3 General population: 20	[159, 161, 163]	[50, 53, 54, 77, 87, 90, 92, 96, 98, 100, 103, 110, 111, 121, 122, 136, 144, 147, 148, 151]
**Mixed**	Older adults: 1 General population: 5	[157]	[32, 86, 104, 109, 143]
**Negative**	General population: 5	–	[108, 118, 134, 139, 152]

### General study characteristics

#### Publication year

Most of the studies focusing on both older adults and the general population were published in the last decade, and the number of publications in both groups increased remarkably in this period (Table 1). The oldest publications meeting our criteria dated from 2007 among studies focusing on older adults, and from 2005 among general population-focused studies.

#### Journal field

More than half of the studies in both literature groups were published in health-related journals (Table 1), followed by inter- or multi-disciplinary journals, transportation or urban studies, and environment- or geography-related journals.

#### Geographical context

The most used settings in walkability studies among both older adults- and general population-focused publications were the US and Canada (50 and 55.7% respectively) (Table 1). This was followed by Europe in both groups (29.2 and 20.5% respectively). However, among publications focusing on older adults, 16.7% were conducted in the Middle East and Asia, while the share among general population-focused literature was only 3.3%. The third most used setting among studies focusing on the general population was Oceania with 17.2% of the studies included in this group, while only one study focusing on older adults was conducted in this geographical context with a share of 4.2%.

### Characteristics of the study design

#### Research design

Most studies in both groups of literature were designed as cross-sectional (75% among older adults- and 86.1% among general population-focused studies) (Table 1). Among publications focusing on older adults the share of longitudinal studies showed a higher percentage (20.8%) compared to that among general population-focused publications (13.1%).

#### Spatial data collection method

The vast majority of studies focusing on both older adults (91.7%) and the general population (93.4%) used GIS to collect their spatial data (Table 1). The share of audit usage among older adults-focused studies (8.3%) was slightly higher compared to the share among studies focusing on the general population (6.6%).

#### Outcome data collection method

Most of the outcome data was collected by self-reports in both literature groups (Table 1). Among studies focusing on the general population, device usage showed a higher share (25.4%) compared to the share among publications focusing on older adults (12.5%). However, using mixed methods to collect walking-related outcome data presented a higher share among older adults-focused studies (29.2%).

### Characteristics of the walkability measures

#### Operationalization of walkability

Studies mostly used indexes to operationalize walkability among older adults and the general population, with a higher share among the latter (79.2 and 91.8%, respectively) (Table 1). The share of using separate variables, however, was higher among studies focusing on older adults (20.8%) compared to the share among the general population literature (8.2%). The most used indexes among older adults-focused publications were the walkability index of Frank et al. (2010) [58], the WalkScore index, and the walkability index of Frank et al. (2005) [59], respectively (data not shown). Among studies focusing on the general population the most preferred index was WalkScore. This was followed by the walkability index of Frank et al. (2010) [58], and new indexes created by the publications.

#### Walkability variables used

In 24 publications focusing on older adults, a total of 167 walkability variables were used. Most of the variables in this group of publications (33.5%) related to land use characteristics (Table 2). This was followed by safety from traffic category with 12%. Following this, street connectivity (11.4%), street design (11.4%), and activity and destination density (10.8%) were the next most used categories of walkability variables among publications focusing on older adults. Population density presented a share of 9%, while greenery and safety from crime each formed 4.8%, respectively, of the publications in this group. Variables related with transportation accessibility and topographic characteristics were the least preferred, while no variables related to socioeconomic characteristics were used among older adults-focused publications.

Among publications focusing on the general population (*n* = 122), a total of 518 variables were used to measure walkability. The most common variables were related to street connectivity (22.4%), and activity and destination density (17.8%). Following these, 16.6% of the walkability variables used in this literature group were related to population density, and 16.4% to land use characteristics. Variables related to street design formed 10.6%, while safety from traffic had 5% share. Greenery (3.9%), transportation accessibility (3.1%), safety from crime (2.7%), topographic (1.2%) and socio-economic characteristics (0.4%) were also used but to a lower extent compared to other categories among this group of publications.

See Supplementary Material, Table S2. for walkability variables used in each study.

### Spatial extent and unit

#### Spatial extent

All publications on older adults focused on residential areas to measure walkability (Table 1). Among publications on the general population, residential areas were also the most preferred spatial extent with 87.7%. Notwithstanding, 4.1% of the publications in this group used school sites, while some had more than one spatial extent in their studies such as residential and workplace or residential and school site. Lastly, other spatial extents such as daily walking itineraries or routes to parks were also used among studies on the general population.

#### Spatial unit

Most of the publications in both groups used buffers to measure walkability in their studies (Table 1). This was followed by statistical units (e.g., census block groups, statistical areas/sectors/tracts, etc.) (16.7% among older adults-focused publications and 20.5% among the general population literature) and administrative units (e.g., zip/postal codes, neighborhood boundaries, etc.) (12.5 and 7.4%, respectively).

#### Buffer type and size

Among 17 older adults-focused studies using buffers, 53% used street network buffers while the rest used circular buffers (Table 3). Some studies used more than one buffer size in this group of publications. Among the 20 buffer sizes used, the most common were 1000 m and 2500 m (25% each) (Table 3). This was followed by 400 m- and 500 m-buffers, each presenting 15% of the total. Buffers equal to or less than 1000 m were preferred more (65%) than those greater than 1000 m in this group (35%) (See Supplementary Material, Section 1.4.3 for detailed information on the selection of 1000 m as a threshold).

Among 82 papers using buffers in publications focusing on the general population, a total of 91 buffer types were used. Among these, 51.6% were street network buffers, 46.2% were circular buffers, and 2.2% were sausage buffers. Similar to older adults-focused publications, some of the papers focusing on the general population used more than one buffer size in their studies. Among the total of 125 buffer sizes used, buffers less or greater than 1000 m were almost equally preferred among the publications in this group. Due to the high usage of the WalkScore index among the publications focusing on the general population, the most common buffer size was 2500 m (≈1.5 miles) (30%) (See Supplementary Material, Section 1.4.3 for buffer sizes of WalkScore indexes). This was followed by 1000 m (25%), 1600 m (≈1 mile) (11%,) and 800 m (≈0.5 mile) (9%).

### Associations found between walkability and walking-related outcomes

Most of the publications focusing on both older adults (62.5%) and the general population (60.7%) found positive associations between walkability and walking-related outcomes (Table 1). One fifth of the publications on older adults found no association while the share was lower among papers focusing on the general population (14.8%). There was no paper with a negative association in the literature on older adults, whereas 4.1% of the publications on the general population found negative associations between walkability and walking-related outcomes. Partial associations were found among 12.5% of studies focusing on older adults, while this proportion was 16.4% among studies on the general population.

## Discussion

Understanding, defining and/or measuring walkability is essential for creating more democratic, sustainable, and healthy environments. These benefits are particularly important for older adults, for whom walking is one of the easiest ways to achieve the recommended daily physical activity levels. Therefore, a review of the operationalization of objective walkability, how it related to walking outcomes, and how this relationship differed for older adults compared to the general population, could shed light to the gaps in the literature and thus be useful for academics interested in this field of research, as well as being insightful for urban designers, planners, and decision makers to create more inclusive places that consider the differences of individuals and settings.

In our results, the increase in the number of walkability studies in the last decade is promising. This applies for studies focusing on older adults but also the general population, as mentioned in previous reviews [20, 27, 28]. However, the fact that in our review many walkability studies in both groups focused on similar geographic settings (the US, Canada, and Europe) is of concern in terms of generalization of the results, despite the higher proportion observed in the number of studies focusing on older adults conducted in the Middle East and Asia. As it was also highlighted in previous studies, translating findings from these most common settings could be misleading, considering the differences in morphologies and land-use configuration between urban contexts across the globe, and the high proportion of studies conducted in high-income countries [12, 20, 26]. For this reason, the literature also lacks examples from middle- and low-income countries or cities, where walking is not only an important and a low-cost type of PA for a healthier life, but also one of the most accessible ways of transportation [3]. To this end, instead of following a one-size-fits-all approach, more studies conducted in different countries and even different cities of the same country, would bring new perspectives to walkability studies by highlighting the differences among settings, and their relationship with walking behavior of the general population and older adults in particular.

In terms of research design, cross-sectional studies were the most common among both groups of literature, as was also previously pointed out by other reviews [27–29, 178]. However, compared to studies focusing on the general population, it is promising that we observed a higher ratio of longitudinal studies among older adults-focused publications in our review. As is suggested in studies of aging, longitudinal designs are essential to understand complicated relationships among events or risks and outcomes, as well as to reduce possible biases, such as selection bias in sampling [179]. Thus, more longitudinal studies focusing on older adults would bring a more comprehensive understanding of walkability for this age group in the future, besides providing more reliable results.

In terms of data collection, most of the analyzed studies focusing on older adults used self-report measures to obtain walking-related outcomes. The ratio of use of technological devices for outcome data collection among older adults-focused studies presented a lower proportion than that of studies focusing on the general population, as was also mentioned in previous reviews [28, 178]. This could be due to the methodological challenges of these devices to capture older adults’ mobility, such as low battery life, underestimation of PA due to body-placement of the device, or difficulties encountered by participants when using these devices [180]. However, self-report measures have various disadvantages, especially among studies focusing on older adults, such as not capturing all daily activity patterns since individuals may not consider some activities, like dancing, as a type of PA, possibility of changes in older adults’ health status and/or mood, or problems with memory and cognition that could affect accurate recall of PA on a survey [181]. Thus, as previous studies suggested, the optimal reliability of results, especially in older adults’ mobility research, could be gathered from the use of objective or mixed methods [182, 183], which promisingly presented a higher proportion among older adults-focused papers included in our review.

Regarding the operationalization of walkability, most studies in both groups used indexes. The most preferred index in studies focusing on the general population was WalkScore, as it was also mentioned [21], and criticized previously for being an “insufficient metric for population health studies”, and for not capturing “the experiential nature of walking nor walkability” since it excludes recreational walking ( [14], pp. 3, 8), as well as the lack of consideration of attributes that would contribute to walking [20], such as measures related with safety [13]. Also, this index is only validated in the US and Canada, as stated on their website. Thus, its use in other settings could be highly misleading. Among studies focusing on older adults, the most common index was the walkability index of Frank et al. (2010) [58]. Although the variables used in this index (net residential density, retail floor area ratio, land use mix, and intersection density) could be meaningful to some extent to measure walkability in some other settings, this study was based on the data from two US cities and was not created specifically for older adults. Thus, although the values assigned for each variable in the formula were modified for adaptation in some of the reviewed papers [154, 177], its use to study this age group and in different settings could also be misleading. Although walkability differed widely for older adults compared to other pedestrians, as highlighted in an empirical research [12] as well as in a recent study proposing a “walkability index for elderly health” [184], in our review, the proportion of publications among older adults-focused studies creating their own indexes was lower than that among publications focusing on the general population. However, the ratio of the usage of separate variables was higher among older adults-focused studies. Depending on the selection of variables, this measure could provide more meaningful results for walkability and its relationship with older adults’ walking, rather than using an index which was not designed specifically for this age group. We believe that by using more specific variables or indexes, not only for the age groups under study but also for the settings, walkability measures could become more precise, and this would help create more walkable areas and promote walking for all.

The most used category of walkability variables among studies focusing on older adults were those related to land use characteristics in our review. This was followed by variables related to safety from traffic, which are intuitively believed to be specifically relevant to older adults' walking and used widely, as stated in a previous review [26]. The ratio of the usage of variables related with street design was similar for publications focusing on older adults and the general population. Although this category included variables found by previous studies to be specifically essential for older adults’ walking, such as sidewalk availability/ width/ material [185], presence of benches [186, 187] or restrooms [28] plus many other examples, usage of these variables did not present considerably higher proportions among publications focusing on older adults. The ratio of using greenery-related variables was slightly higher among studies focusing on older adults compared to the general population. This is perhaps due to the numerous studies in the literature highlighting the positive relationship found between the presence of green areas (including parks or street trees) and older adults’ walking [188–190]. However, the proportion of using these variables among older adults-focused studies was still low compared to the importance of this variable for their PA.

Regarding the spatial extent for measuring walkability, different from publications on the general population, studies on older adults focused only on residential areas in their research. Similarly, the most used spatial units among studies on older adults were buffers which are equal to or smaller than 1000m, and administrative units (e.g., neighborhood boundaries/ units, zip/postal codes, etc,) presented a higher share among this group of literature. These results were expected considering that the range of activity among older adults mostly decreases to the immediate vicinity of their residences [191], and doubtless this sheds more importance on the characteristics of the built environment in the neighborhoods. However, this also limits the range of walkability studies by underestimating individual differences, since not all older adults’ activity range or levels are the same. Additionally, different types of walking, such as recreational walking, could take place farther than the residential areas, and limiting studies with these extents could easily exclude these types of walking [192]. Thus, more studies using wider spatial extents and units in the future would provide more detailed information on walkability and its relationship with different types of walking, settings, and individuals.

Finally, most of the papers included in our review found a positive association between walkability and walking-related outcomes in both groups of publications. Among studies focusing on older adults, publications which found a positive association, as well as those which did not find any associations showed a higher proportion compared to publications focusing on the general population. The higher proportion of the latter could be explained by the lack of age-specific index usage among publications focusing on older adults [184]. Using indexes which are not created while considering specific needs of this age group could be limited or even misleading in understanding the relationship between walkability and walking. Regarding the high percentage of positive associations found between walking and walkability, previous reviews also presented similar results [28, 29]. A review on the general population explained the reason for this as the high number of studies conducted in high-income countries, since these settings are less likely to have deficiencies in the built environment, such as poor sidewalk infrastructures, or safety issues, such as high crime rates, compared to middle- or low-income settings [20, 193]. Thus, more studies conducted in different countries and even in different cities of a country, especially taking into consideration the possible socioeconomic differences between cities, would bring a wider perspective to the research on walkability, be helpful to overcome the uncertainties in the literature, as well as inform governments to create solutions for creating more walkable places for various population groups, and promoting walking in settings with different characteristics.

### Strengths and limitations

The first strength of our systematic review is that it focuses particularly on walkability studies. Second, it provides results for older adults and the general population separately, which highlights the differences more and helps to find solutions for creating better environments for everyone. Third, this review includes only objective operationalization of walkability. Considering the main focus on older adults, this is accepted as one of the most precise methods [12, 18, 182]; thus we believe that the studies included in this review provided high reliability results. Finally, this review provided comprehensive information about not only how objective walkability has been defined and measured, which could be insightful for governments, but also scrutinized the methodologies used in walkability studies, which could be useful for researchers interested in conducting both literature reviews and empirical analyses on walkability. However, this systematic review is not exempt from limitations. First, although we included numerous characteristics of the studies in the content analysis, we did not cover other characteristics, such as sample size, which could provide different insights. Future research could consider including this variable in their reviews to enrich the body of literature. Second, selecting papers published only in English could have resulted in a language-based bias. However, we believe that the reviewed studies and the analysis presented here are representative, since the majority of empirical studies worldwide are published in English [194]. Finally, as mentioned in other reviews [13, 195] other types of biases, such as spatial selection bias (e.g., residential selection bias, whether people who walk more choose to live in highly walkable areas), or recall bias (e.g., studies using self-reported PA) among the included studies could have impacted their results and thus the results of our review and our interpretations, even indirectly.

## Conclusion

This review draws attention to how objective walkability has been operationalized, how it is related to walking outcomes, and how these differed among studies focusing on older adults and the general population. Despite the promising increase in the last decade in the number of publications focusing on walkability for all sorts of population groups, the literature still lacks studies 1) focusing on different settings, especially low- and middle-income settings, 2) using wider spatial extents rather than only neighborhood scale, 3) using longitudinal designs, 4) using objective or mixed methods to collect their outcome data related with walking, and 5) creating indexes or using separate variables which are specific for settings and population groups, such as older adults. With future studies aiming to address these points, walkability studies could become more comprehensive and provide better answers to urban design and planning problems.

The methodologies used and the gaps found in the walkability literature highlighted in this review could be useful for researchers to conduct future reviews, as well as empirical analyses on walkability. Additionally, the differences in the definition and operationalization of objective walkability for older adults versus the general population summarized in this study could be insightful for not only researchers interested in the field, but also urban designers, planners, or local governments aiming to create more walkable places that would meet the needs of most population groups, but specifically older adults’, in different settings. These would enrich the walkability literature and contribute to more democratic, sustainable, and healthy environments, as well as the societies in general.

## Supplementary Information

**Additional file 1.**

## Data Availability

The Table 4 in manuscript and Table S2 in supplementary material, could be used for any related purposes required here (e.g., replication, interpretation). When analyzing publications included in the review, we also gathered other types of information which are not presented in this manuscript. However, original complete datasets could also be provided on request. For this, please contact corresponding author.
