# Supplementary material for: How different are objective operationalizations of walkability for older adults compared to the general population? A systematic review
Source: BMC Geriatr. 2022 Aug 15;22:673. doi: 10.1186/s12877-022-03233-x (PMC9377138; doi:10.1186/s12877-022-03233-x)
Supplement: Supplementary file 1 — Additional file 1. [file 12877_2022_3233_MOESM1_ESM.docx]

**SUPPLEMENTARY MATERIAL**

# Categorizations and codings

## General study characteristics

## Publication year

Publication years were grouped under 5-year periods (2005-2010, 2011-2015), except the last period (2016-2019) which consisted of 4 years.

See Table 4 in the manuscript for publication period of each publication included in the analysis.

## Journal fields

Journals were categorized depending on the focus field mentioned in their own websites as: health-related, inter- or multi-disciplinary studies, transportation or urban studies, and environment- or geography-related journals **(Table S1)**.

See Table 4. in the manuscript for journal field of each publication included in the analysis.

**Table S1. List of journals under each journal field**

| Journal field and name of the journals | # of publications |  | Journal field and name of the journals | # of publications |
| --- | --- | --- | --- | --- |
| Health | 90 |  | Environment or Geography | 4 |
| The International Journal of Behavioral Nutrition and Physical Activity | 14 |  | Environment and Behavior | 2 |
| Preventive Medicine | 9 |  | Geographical Research | 1 |
| American Journal of Preventive Medicine | 7 |  | Transactions in GIS | 1 |
| Journal of Physical Activity & Health | 7 |  |  |  |
| BMC Public Health | 5 |  | Journal field and name of the journals | # of publications |
| Journal of Urban Health : Bulletin of the New York Academy of Medicine | 4 |  | Transportation or Urban Studies | 5 |
| American Journal of Health Behavior | 2 |  | International Journal of Sustainable Transportation | 1 |
| American Journal of Health Promotion : AJHP | 2 |  | Journal of the American Planning Association | 1 |
| American Journal of Public Health | 2 |  | Journal of Transport and Land Use | 1 |
| BMJ Open | 2 |  | Journal of Urban Planning and Development - ASCE | 1 |
| Journal of Environmental and Public Health | 2 |  | Transportation Research Part D: Transport and Environment | 1 |
| Journal of Immigrant and Minority Health | 2 |  |  |  |
| Preventing Chronic Disease | 2 |  | Journal field and name of the journals | # of publications |
| Appetite | 1 |  | Inter- or Multi-disciplinary Studies | 47 |
| Blood Purification | 1 |  | International Journal of Environmental Research and Public Health | 13 |
| BMC Geriatrics | 1 |  | Health & Place | 11 |
| British Journal of Sports Medicine | 1 |  | Journal of Transport and Health | 8 |
| Canadian Journal of Public Health | 1 |  | Social Science & Medicine | 5 |
| Canadian Journal on Aging = La Revue Canadienne Du Vieillissement | 1 |  | PloS One | 3 |
| Cancer Epidemiology, Biomarkers & Prevention | 1 |  | Medicine and Science in Sports and Exercise | 2 |
| CMAJ Open | 1 |  | Environment and Planning B: Urban Analytics and City Science | 1 |
| Disability and Health Journal | 1 |  | Nature | 1 |
| Environmental Health Perspectives | 1 |  | Sustainability | 1 |
| European Journal of Public Health | 1 |  | International Journal of Health Geographics | 1 |
| Health Reports | 1 |  | Journal of Human Kinetics | 1 |
| International Journal of Public Health | 1 |  |  |  |
| Journal of Aging and Health | 1 |  |  |  |
| Journal of Community Health | 1 |  |  |  |
| Journal of Environmental Health | 1 |  |  |  |
| Journal of Epidemiology and Community Health | 1 |  |  |  |
| Journal of Obesity | 1 |  |  |  |
| Journal of Public Health | 1 |  |  |  |
| Lancet (London, England) | 1 |  |  |  |
| Malaysian Journal of Nutrition | 1 |  |  |  |
| Obesity Surgery | 1 |  |  |  |
| Pediatric Exercise Science | 1 |  |  |  |
| Population Health Metrics | 1 |  |  |  |
| Preventive Medicine Reports | 1 |  |  |  |
| Public Health | 1 |  |  |  |
| Research Quarterly for Exercise and Sport | 1 |  |  |  |
| Risk Management and Healthcare Policy | 1 |  |  |  |
| Sports Medicine (Auckland, N.Z.) | 1 |  |  |  |
| Supportive Care Cancer | 1 |  |  |  |

## Geographical context (study setting)

Study settings were sorted under five groups: 1) Europe (including Austria, Belgium, Czech Republic, Denmark, Finland, France, Germany, Ireland, Portugal, Spain, Sweden, and the UK), 2) Middle East and Asia (including Iran, Japan, Malaysia, Singapore, South Korea, and Taiwan), 3) Oceania (including Australia and New Zealand), 4) Latin America (including Brazil and Mexico), and 5) the United States and Canada. Besides, there were two studies [1, 2] conducted their analyses on multiple countries.

See Table 4 in the manuscript for study setting of each publication included in the analysis.

## Demographic groups under study

We created four categories for demographic groups under study according to how they were defined in the original study as: all population, adults, young people, and older adults. The definition of adults varied vastly across studies as being older than a specific age without defining a maximum limit (e.g., older than 16, 18, 20, 25 or 45 years) or being within specific age intervals (e.g., 20-64, 20-70, 37-73, or 18-90 years). Under the young people group we included studies focusing on students (e.g., [3, 4]), adolescents (e.g., [5, 6]), children (e.g., [7, 8]), and toddlers (9). Most of the included studies defined older adults as people ≥ 65 years (besides a few that put the age limit at 70 or 75 years). There was one study that defined older adults as being over 55 years (10), and it was included under the older adults group in our analysis.

In cases when the papers focused on specific groups (SG) without any information on age, like “cancer survivors” (11) or “anonymous participants from activity-oriented mobile phone application” (12), then we included them under the “all population” category. However, when they defined an age group among the SG, such as “female adults with Body Mass Index between 21-39.9” (13), “healthy recent Cuban immigrants between 30-45 years” (14), or “employed adults” (15) then we included these in the related age group categories (in these cases in the adults group).

See Table 4 in the manuscript for demographic groups under study in each publication included in the analysis.

## Characteristics of the study design

## Research design

Studies providing results from both cross-sectional and longitudinal data were coded as “mixed”. See Table 4 in the manuscript for research design of each publication included in the analysis.

## Spatial data collection method

We have only included publications using objective methods to operationalize walkability in this review. Studies using Geographic Information Systems (GIS) or environmental/ street audits for spatial data collection were coded accordingly either as “GIS” or “audit”.

See Table 4 in the manuscript for spatial data collection method of each publication included in the analysis.

## Outcome data collection method

Studies using objective methods to collect their outcome data (e.g., accelerometer, GPS devices) were coded as “device” and the ones using subjective methods (e.g., interviews, travel diaries) were coded as “self-reported”. Those using both methods were coded as “mixed”.

See Table 4 in the manuscript for outcome data collection method of each publication included in the analysis.

## Characteristics of walkability measures

## Operationalization of walkability

Publications were coded under “index” category when they 1) used one existing index or a combination of many (e.g., the walkability index of Frank et al. (2010) (16), WalkScore…), 2) created their own indexes, or 3) used separate variables and presented their results as a score. If a study used separate variables or an environmental audit, and provided results separately for each variable, then it was coded under the “separate variables” category.

See Table 4 in the manuscript for the operationalization of walkability in each publication included in the analysis.

## Walkability variables used

Mostly, studies used more than one variable to measure walkability and each variable was grouped under the relevant category. All variables used in indexes or environmental/street audits (sometimes more than 80 variables in a single audit) were coded one by one under each corresponding category for a detailed understanding of operationalization of walkability.

Variables used in studies were grouped under eleven categories as 1) Population density, 2) Activity and destination density/ access to services, 3) Socioeconomic characteristics, 4) Land use characteristics, 5) Street connectivity, 6) Topographic characteristics, 7) Street design, 8) Safety from traffic, 9) Safety from crime, 10) Transportation accessibility, and 11) Greenery.

Variables were included in the abovementioned categories as they were used in the original study or according to their relevance. When the original study included a variable under a category which is similar to ours, then the categorization was done accordingly. However, this was not the case all the time. For instance, variables such as presence of shopping mall, schools, restaurant, fitness center, religious institution, post office, etc. used in the Pedestrian Environment Data Scan (PEDS) audit would have been included under “activity and destination density/access to services” category in our analysis, however in the original study (17) these were related to and included under the land use category, so we also have included them under “land use characteristics” category in our study. Similarly in the same audit, cul-de-sac or permanent street closing was included under “safety from crime” category in the original study and thus in our analysis, although other variables related to cul-de-sac/ dead-end streets from other studies were included under “street connectivity” category.

Lastly, WalkScore and StreetSmart WalkScore indexes were coded under “activity and destination density/ access to services” category since both of them were mainly based on the distance to destinations/ amenities, while TransitScore index was coded under “transportation accessibility” category due to its specific focus.

See Table S2. for walkability variables included under each category and used in each publication included in the analysis.

## Spatial extent and unit

## Spatial extent

Spatial extents used in studies were coded as they were mentioned in the original publication (e.g., residential, school site or workplace). Studies using other spatial extents such as daily walking itineraries, entire cities, or routes to parks were coded under “other” category.

See Table 4 in the manuscript for spatial extent used in each publication included in the analysis.

## Spatial unit

For spatial units, studies used buffers, administrative units (e.g., postal codes, municipal levels, neighborhood boundaries/units, etc.) and statistical units (e.g., census groups, statistical areas/ districts/ sectors, etc.). Studies using units such as street segments, country level, or enrollment zones were coded as “others”. Finally, studies using more than one spatial unit (e.g., buffer+ census block group) were coded as “combination”.

See Table 4 in the manuscript for spatial extent and unit used in each publication included in the analysis.

## Buffer type and size

When studies used more than one buffer type or size (e.g., for sensitivity analysis), each was coded separately under the corresponding group. Buffer distances provided in studies as miles were converted to meters (1 mile=1,609.34 m) and included in the closest group (e.g., studies using 0.5 miles were included in the group of 800 m). Unless otherwise specified, studies using WalkScore (including Street Smart and Transit WalkScore) were accepted to be using 2,500 m (≈1.5 miles) buffers as it was stated in the methodological information provided in their official website (https://www.walkscore.com/methodology.shtml). Regarding their buffer types, unless otherwise specified, those using WalkScore and TransitScore were coded as circular buffers, and those using StreetSmart WalkScore were coded as street network buffers depending on the information provided in their aforementioned website.

For a better interpretation of the results, we also grouped buffer sizes into two groups as: 1) equal to and less than 1,000 m, and 2) greater than 1,000 m, depending on the findings of previous studies stating that older adults’ physical activity (PA) was related to walkability within 1,000 m buffers [18, 19] and to other built environment features in smaller buffer sizes (20).

See Table 4 in the manuscript for buffer types and sizes used in each publication included in the analysis.

## Associations found between walkability and walking

For studies using indexes/scores, the associations were mostly clear and coded accordingly as positive, negative, or no association. When studies provided results for each walkability variable separately and when the difference between the number of associations found between walkability variables and outcomes was greater than one (e.g., three positive, and one negative association), then the majority defined the final decision for that paper as either positive, negative, or no association (in this case it was positive). In the cases where the difference is not clear (e.g., three positive, three negative, and two no association) then they were coded as partial. Additionally, studies providing different results for different buffer sizes (e.g., for sensitivity analysis) or different walking-related outcomes (e.g., transportation walking, leisure walking…) were also coded as “partial”. Finally, studies presenting results for different population subgroups (e.g., female vs. male) or for different settings in the study (e.g., different cities in a country) were coded as “mixed”.

See Table 4 in the manuscript for associations found between walkability and walking-related outcomes in each publication included in the analysis.

**Table S2. Walkability variables used in analyzed publications focusing on older adults vs. general population**

| Walkability variables | Reference |
| --- | --- |
| **POPULATION DENSITY** |  |
| Population density | Older adults  (21,22)  General population  (15,23–37) |
| Residential density | Older adults  (10,18,38–46)  General population  (2,4,47–56,5,57–66,6,67–76,7,77–86,8,87–96,11,97–100,13,16,23) |
| Household density | Older adults  (101)  General population  (102) |
| Residential dwelling density | General population  (32,103,104) |
| More dwelling units per acre of the parcel | Older adults  (19) |
| Single family residential (SFR) parcel count | General population  (105) |
| Acres of SFR land | General population  (105) |
| **ACTIVITY AND DESTINATION DENSITY/ ACCESS TO SERVICES** |  |
| Job/ business density | General population  (26,35,66) |
| SFR parcels less than 4 miles to commercial use | General population  (105) |
| Fast food outlets | General population  (4) |
| More grocery store restaurant or retail clusters in 1km buffer | Older adults  (19) |
| Fewer grocery stores or markets in 1km buffer | Older adults  (19) |
| Local destinations | General population  (75) |
| Neighborhood destinations | General population  (99) |
| Destination accessibility | General population  (27,79) |
| Retail floor area ratio | Older adults  (18,38,39,41–43,101)  General population  (5,16,64,68,70–72,75,78,81,82,84,37,85–89,92,98,102,103,47,51,53,55,59,62,63) |
| Access to retail areas | General population  (24) |
| Shopping malls | General population  (4) |
| Acres of commercial land | General population  (105) |
| Number of shopping malls in 1km buffer | General population  (106) |
| Total floor area of shopping mall in 1km buffer | General population  (106) |
| Recreation facilities | General population  (4) |
| Private recreation density | Older adults  (39) |
| Number of private recreational facilities | General population  (5) |
| Density of mix of recreational facilities /km2 | General population  (35) |
| Mix of recreational facilities | General population  (26) |
| Fitness facility density | General population  (66) |
| Distance to school | General population  (79) |
| Smaller size of closest office complex | Older adults  (19) |
| Longer distance to closest office mixed use complex | Older adults  (19) |
| Fewer educational parcels in 1km buffer | Older adults  (19) |
| WalkScore | Older adults  (107,108)  General population  (1,12,102,109–117,13,118–127,14,128–130,15,27,28,31,33,84) |
| StreetSmart Walkscore | Older adults  (131–133)  General population  (115,134–137) |
| Market concentration/ market monopoly | General population  (30) |
| Stores within easy walking distance from home – Irvine-Minnesota Inventory (IMI audit) | General population  (138) |
| Many places to go within easy walking distance from home- IMI audit | General population  (138) |
| Employment density | General population  (23) |
| Retail service / job density | General population  (23) |
| **SOCIOECONOMIC CHARACTERISTICS** |  |
| Residential property values | General population  (66)v |
| Socioeconomic status | General population  (79) |
| **LAND USE CHARACTERISTICS** |  |
| Residential percentage | General population  (9) |
| Land use mix | Older adults  (10,17,43–46,101,18,21,22,38–42)  General population  (2,4,29,32–34,36,37,47–50,5,51–55,57–61,6,62–65,67–72,7,73–78,80–83,11,84–93,13,94–100,102–104,15,138–140,16,25) |
| Land use intensity | Older adults  (38) |
| Walkable land use mix | Older adults  (21) |
| Number and variety of land uses | Older adults  (141) |
| Proportion of mixed land use | General population  (32) |
| Land use mix and diversity | General population  (8,50) |
| Single family home detached - Pedestrian Environment Data Scan (PEDS audit) | Older adults  (17)  General population  (13) |
| Single family home duplex semidetached -PEDS audit | Older adults  (17) |
| Town house terrace row house- PEDS audit | Older adults  (17) |
| Flat apartments more than 3 stories- PEDS audit | Older adults  (17)  General population  (13) |
| Mobile homes caravan parks cabins- PEDS audit | Older adults  (17)  General population  (13) |
| Post office police station courthouse- PEDS audit | Older adults  (17) |
| Hospital medical facility- PEDS audit | Older adults  (17) |
| Retail shops restaurants- PEDS audit | Older adults  (17)  General population  (13) |
| Office institutional- PEDS audit | General population  (13) |
| Hotel hospitality- PEDS audit | Older adults  (17) |
| Industrial area- PEDS audit | General population  (13) |
| Vacant undeveloped areas- PEDS audit | General population  (13) |
| Gas service station- PEDS audit | Older adults  (17) |
| Big box shop- PEDS audit | Older adults  (17) |
| Shopping mall- PEDS audit | Older adults  (17) |
| Strip mall row of shops- PEDS audit | Older adults  (17) |
| Plaza square park playground landscaped open space- PEDS audit | Older adults  (17) |
| Public space other- PEDS audit | Older adults  (17) |
| Gym Fitness center- PEDS audit | Older adults  (17) |
| Movie theatre- PEDS audit | Older adults  (17) |
| Recreational other- PEDS audit | Older adults  (17)  General population  (13) |
| Harbor marina- PEDS audit | Older adults  (17) |
| Nature feature- PEDS audit | Older adults  (17) |
| Open field golf course- PEDS audit | Older adults  (17) |
| Lake pond- PEDS audit | Older adults  (17) |
| Stream river canal creek- PEDS audit | Older adults  (17) |
| Ocean beach- PEDS audit | Older adults  (17) |
| Mountain Hills- PEDS audit | Older adults  (17) |
| Community center library- PEDS audit | Older adults  (17) |
| Museum auditorium concert hall theater- PEDS audit | Older adults  (17) |
| Religious institution- PEDS audit | Older adults  (17) |
| Art or craft galleries- PEDS audit | Older adults  (17) |
| Restaurants- PEDS audit | Older adults  (17) |
| Coffee shops- PEDS audit | Older adults  (17) |
| Corner store- PEDS audit | Older adults  (17) |
| Primary middle or junior high school- PEDS audit | Older adults  (17) |
| Highschool- PEDS audit | Older adults  (17) |
| School other- PEDS audit | Older adults  (17) |
| Day care center- PEDS audit | Older adults  (17) |
| Vertical mixed use- PEDS audit | Older adults  (17) |
| Parking structure- PEDS audit | Older adults  (17) |
| **STREET CONNECTIVITY** |  |
| Three way intersection density | General population  (32) |
| Four way intersection density | General population  (32) |
| Intersection count | General population  (90,105) |
| Street density | Older adults  (142)  General population  (23,29) |
| Street connectivity /intersection density | Older adults  (10,18,45,46,101,142,22,38–44)  General population  (2,4,27,28,30–34,36,37,47,5,48–57,7,58–64,67–69,8,70–79,9,80–89,11,91–100,13,102–104,143,144,16,25) |
| Road-based intersection density | General population  (55) |
| Connectivity | Older adults  (145)  General population  (143) |
| Street intersection | General population  (15,65,66) |
| Connected node ratio | General population  (9,37,146) |
| Miles of roads | General population  (105) |
| Average block area | General population  (50) |
| Average block length | General population  (9,37) |
| Block length | General population  (143) |
| Smaller size of block where residence is located | Older adults  (19) |
| Path cycleway length | General population  (26,35) |
| Road density | General population  (24,146) |
| Number of cul-de-sacs | General population  (146) |
| Cul-de-sac density | General population  (65) |
| Number of junctions | General population  (146) |
| Segment has dead-end- PEDS audit | General population  (13) |
| Segment continues- PEDS audit | General population  (13) |
| Road dead-ends but path continue- PEDS audit | General population  (13) |
| Footpath based intersection density | General population  (55) |
| Path continuity- SPACES audit | General population  (99) |
| Sidewalk completeness continuity- PEDS audit | General population  (13) |
| Sidewalks continuity to other sidewalks crosswalks- PEDS audit | General population  (13) |
| Direct route- SPACES audit | General population  (99) |
| Short distance between intersections- IMI audit | General population  (138) |
| Presence of many alternative routes for getting from place to place -IMI audit | General population  (138) |
| Few or no cul-de-sacs streets- IMI audit | General population  (138) |
| Area of neighborhood including in 1km buffer | General population  (106) |
| Ratio of three or four way intersections | General population  (23) |
| Total length of retaining wall of apartment complex | General population  (106) |
| Presence of alley- IMI audit | Older adults  (17) |
| Pedestrian access point through cul-de-sac- IMI audit | Older adults  (17) |
| **TOPOGRAPHICAL CHARACTERISTICS** |  |
| Land slope sloping streets | General population  (31) |
| Hills | General population  (139) |
| Flat area- PEDS audit | General population  (13) |
| Slight hill- PEDS audit | General population  (13) |
| Steep hill- PEDS audit | General population  (13) |
| Gradient steepness- SPACES audit | General population  (99) |
| Steep slope- PEDS audit | Older adults  (17) |
| **STREET DESIGN** |  |
| Road conditions materials uniformity- PEDS audit | General population  (13) |
| Path obstructions- PEDS audit | General population  (13) |
| Parking on and off street- PEDS audit | General population  (13) |
| Pedestrian amenities | General population  (13,75) |
| Way finding aids- PEDS audit | General population  (13) |
| Sidewalk density | General population  (9,37,66) |
| Sidewalk length | General population  (26,106) |
| Sidewalk availability | General population  (140) |
| Presence and width of sidewalks | Older adults  (141) |
| Sidewalk length density in meters/ km2 | General population  (35) |
| Informal pedestrian network PEDSHED | General population  (3,144) |
| Walkshed area | General population  (26,35) |
| Paved trail- PEDS audit | General population  (13) |
| Sidewalk coverage | Older adults  (145) |
| Footpath density | General population  (146) |
| Effective walkable area | General population  (146) |
| Sidewalk coverage design material | General population  (139,147) |
| Street amenity shady trees streetlamps shops | General population  (13,147) |
| Total length of trails in 1km buffer | General population  (106) |
| Total length of streets with pedestrian sidewalks (m) | General population  (106) |
| Ratio of pedestrian sidewalks | General population  (106) |
| Total length of pedestrian zones | General population  (106) |
| Geometry of street canyons | General population  (12) |
| Footpath- PEDS audit | Older adults  (17)  General population  (13) |
| Footpaths complete on both sides- PEDS audit | Older adults  (17) |
| Benches chairs- PEDS audit | Older adults  (17) |
| Public restroom- PEDS audit | Older adults  (17) |
| Footpath shade- PEDS audit | Older adults  (17) |
| Bus stop with seating- PEDS audit | Older adults  (17) |
| Bus stop without seating- PEDS audit | Older adults  (17) |
| Path type- SPACES audit | General population  (99) |
| Path surface type- SPACES audit | General population  (99) |
| Width/ number of path lane- PEDS audit | General population  (13,99) |
| Width of the street- SPACES audit | General population  (99) |
| Vehicle parking- SPACES audit | General population  (99) |
| Curb type- SPACES audit | General population  (99) |
| Street maintenance | General population  (139) |
| Cleanliness litter - PEDS audit | General population  (13) |
| Articulation in building designs- PEDS audit | General population  (13) |
| Neighborhood maintenance | General population  (139) |
| Interesting sights | General population  (139) |
| Beauty aesthetics | Older adults  (141)  General population  (75,139) |
| Upkeep | General population  (147) |
| General maintenance- PEDS audit | Older adults  (17) |
| Attractiveness of segment- PEDS audit | Older adults  (17) |
| Interesting urban design- PEDS audit | Older adults  (17) |
| Open view- PEDS audit | Older adults  (17) |
| Attractiveness of the view- PEDS audit | Older adults  (17) |
| Path maintenance- SPACES audit | General population  (13,99) |
| Many interesting things to look at while walking- IMI audit | General population  (138) |
| Many attractive natural sights- IMI audit | General population  (138) |
| Attractive buildings homes- IMI audit | General population  (138) |
| Enclosure- PEDS audit | General population  (13) |
| Pleasantness | Older adults  (17) |
| Available street parking, parking availability | Older adults  (141) |
| Other sidewalks greenbelt trails paths- PEDS audit | Older adults  (17)  General population  (13) |
| Access to public space- PEDS audit | Older adults  (17) |
| Pedestrian street closed to cars- PEDS audit | General population  (13) |
| Powerlines along segment- PEDS audit | General population  (13) |
| Bicycle lane- PEDS audit | General population  (13) |
| **SAFETY FROM TRAFFIC** |  |
| Traffic volume | Older adults  (145)  General population  (13,99) |
| Bike or Pedestrian path- PEDS audit | General population  (13) |
| Vehicular traffic exposure | General population  (3,144) |
| Low road speed distance to roads with speed limit | General population  (37) |
| Traffic circle roundabout- PEDS audit | Older adults  (17) |
| Median strip- PEDS audit | Older adults  (17) |
| So much traffic along nearby streets | General population  (138) |
| Usually slow speed of traffic on most nearby streets- IMI audit | General population  (138) |
| Speed limits- PEDS audit | General population  (13) |
| Ratio of high speed roads around schools | General population  (79) |
| Traffic speed- SPACES audit | General population  (99) |
| Traffic safety | Older adults  (17)  General population  (138,139,147) |
| Traffic control devices- SPACES audit | General population  (13,99) |
| Presence of crosswalks and pedestrian signals help walkers cross | General population  (138) |
| Most drivers exceed the posted speed while driving- IMI audit | General population  (138) |
| Street crossing- PEDS audit | Older adults  (17)  General population  (13) |
| White line- PEDS audit | Older adults  (17) |
| Colored line- PEDS audit | Older adults  (17) |
| Pedestrian crossing zebra- PEDS audit | Older adults  (17) |
| Different road surface- PEDS audit | Older adults  (17) |
| Refuge island- PEDS audit | Older adults  (17) |
| Vehicle lanes- PEDS audit | Older adults  (17) |
| Marked midblock crossing- PEDS audit | Older adults  (17) |
| Posted speed limit- PEDS audit | Older adults  (17) |
| Speed bump hump raised crosswalk- PEDS audit | Older adults  (17) |
| Curb bulb out curb extension- PEDS audit | Older adults  (17) |
| Curb cuts in segment- PEDS audit | General population  (13) |
| Traffic signal- PEDS audit | Older adults  (17) |
| Stop sign- PEDS audit | Older adults  (17) |
| Give way sign- PEDS audit | Older adults  (17) |
| Pedestrian activated signal- PEDS audit | Older adults  (17) |
| Pedestrian overpass underpass bridge- PEDS audit | Older adults  (17) |
| Traffic signs at the intersection | General population  (140) |
| Crossings | General population  (139,140) |
| Hard buffer between road and path- PEDS audit | General population  (13) |
| Soft buffer between road and path- PEDS audit | General population  (13) |
| Pedestrian safety | General population  (75) |
| Presence of driveways in the segment- PEDS audit | General population  (13) |
| **SAFETY FROM CRIME** |  |
| Bars nightclubs- PEDS audit | Older adults  (17) |
| Front porch- PEDS audit | Older adults  (17) |
| Prominence of garages- PEDS audit | Older adults  (17) |
| Graffiti- PEDS audit | Older adults  (17) |
| Litter- PEDS audit | Older adults  (17) |
| Well lit streets at night- IMI audit | General population  (138) |
| Pedestrians, bikers easily seen by people in their homes- IMI audit | General population  (138) |
| Presence of high crime rate- IMI audit | General population  (138) |
| Crime rate makes it unsafe to walk at night- IMI audit | General population  (138) |
| Gang activity- IMI audit | General population  (138) |
| Groups of teenagers or adults hanging out causing trouble- IMI audit | General population  (138) |
| House or place you suspect drug dealing occurs- IMI audit | General population  (138) |
| Activity level visible persons from age groups in the segment | Older adults  (141) |
| Other pedestrians | General population  (139) |
| Personal safety | General population  (139) |
| Safety from crime | Older adults  (17) |
| Total crime | General population  (31) |
| Cul-de-sac or permanent street closing- PEDS audit | Older adults  (17) |
| Lighting | General population  (13,140) |
| Must you walk through a parking lot to get to most buildings- PEDS audit | General population  (13) |
| **TRANSPORTATION ACCESSIBILITY** |  |
| Transit access | General population  (140) |
| Number of public transit bus routes | General population  (87) |
| Subway stop density | General population  (25,85) |
| Density of rail transit stops | General population  (27) |
| Public transportation access | Older adults  (145) |
| Density of public transit stations | General population  (7,35,65) |
| Bus stop density | General population  (25,26) |
| Public transportation density | General population  (2) |
| Distance to the nearest transit stop | General population  (2) |
| Transportation infrastructure quality | Older adults  (141) |
| TransitScore | General population  (109,115) |
| Transit stops within easy walking distance from home- IMI audit | General population  (138) |
| Accessibility | Older adults  (17) |
| Transit facilities- PEDS audit | General population  (13) |
| **GREENERY** |  |
| Amount of street greenery | General population  (12) |
| Acres of tree canopy | General population  (105) |
| Greenness Normalized difference vegetation index (NDVI) | General population  (15,29) |
| Number/count of parks | Older adults  (39)  General population  (2,4,5,65,66,87) |
| Park access | General population  (24) |
| Trees and greenery | General population  (139) |
| Distribution of parks greenspaces | Older adults  (145) |
| Proportion of greenspace | General population  (26,35) |
| Public park density | General population  (23)  Older adults  (10) |
| Park type mix | General population  (26) |
| Density of mix of park types/ km2 | General population  (35) |
| Area of parks in neighborhood/ km2 | General population  (106) |
| Ratio of area of parks in neighborhood | General population  (106) |
| Ratio of area of parks in neighborhood including 1 km buffer/ km2 | General population  (106) |
| Park playground- PEDS audit | Older adults  (17) |
| Playing or sport field- PEDS audit | Older adults  (17) |
| Public garden- PEDS audit | Older adults  (17) |
| Forest bush- PEDS audit | Older adults  (17) |
| Street trees- PEDS audit | Older adults  (17) |

**References**

1. Althoff T, Sosič R, Hicks JL, King AC, Delp SL, Leskovec J. Large-scale physical activity data reveal worldwide activity inequality. Nature [Internet]. 2017;547(7663):336–9. Available from: https://www.scopus.com/inward/record.uri?eid=2-s2.0-85025128546&doi=10.1038%2Fnature23018&partnerID=40&md5=7c907e5e76f9817f487f4ece5e81ee5d

2. Sallis JF, Cerin E, Conway TL, Adams MA, Frank LD, Pratt M, et al. Physical activity in relation to urban environments in 14 cities worldwide: A cross-sectional study. Lancet [Internet]. 2016;387(10034):2207–17. Available from: https://www.scopus.com/inward/record.uri?eid=2-s2.0-84961877396&doi=10.1016%2FS0140-6736%2815%2901284-2&partnerID=40&md5=88a30379b8f983d3fa4032b013dba9b8

3. Giles-Corti B, Wood G, Pikora T, Learnihan V, Bulsara M, Van Niel K, et al. School site and the potential to walk to school: The impact of street connectivity and traffic exposure in school neighborhoods. Heal Place [Internet]. 2011;17(2):545–50. Available from: https://www.scopus.com/inward/record.uri?eid=2-s2.0-79952539236&doi=10.1016%2Fj.healthplace.2010.12.011&partnerID=40&md5=116a7445625554c66f0185511745d8e1

4. Hobin E, Leatherdale S, Manske S, Dubin J, Elliott S, Veugelers P. A multilevel examination of factors of the school environment and time spent in moderate to vigorous physical activity among a sample of secondary school students in grades 9-12 in Ontario, Canada. Int J Public Health [Internet]. 2012;57(4):699–709. Available from: https://www.scopus.com/inward/record.uri?eid=2-s2.0-84864050139&doi=10.1007%2Fs00038-012-0336-2&partnerID=40&md5=f1fab57efc2082df14a683066e31ee08

5. Wang X, Conway TL, Cain KL, Frank LD, Saelens BE, Geremia C, et al. Interactions of psychosocial factors with built environments in explaining adolescents’ active transportation. Prev Med (Baltim) [Internet]. 2017;100:76–83. Available from: https://www.scopus.com/inward/record.uri?eid=2-s2.0-85017630623&doi=10.1016%2Fj.ypmed.2017.04.008&partnerID=40&md5=91938048ec32c9fa5066f8205bb4942f

6. De Meester F, Van Dyck D, De Bourdeaudhuij I, Deforche B, Cardon G. Do psychosocial factors moderate the association between neighborhood walkability and adolescents’ physical activity? Soc Sci Med. 2013 Mar;81:1–9.

7. Buck C, Tkaczick T, Pitsiladis Y, De Bourdehaudhuij I, Reisch L, Ahrens W, et al. Objective Measures of the Built Environment and Physical Activity in Children: From Walkability to Moveability. J Urban Heal. 2014 Feb;92(1):24–38.

8. D’Haese S, Gheysen F, De Bourdeaudhuij I, Deforche B, Van Dyck D, Cardon G. The moderating effect of psychosocial factors in the relation between neighborhood walkability and children’s physical activity. Int J Behav Nutr Phys Act [Internet]. 2016;13(1):1–16. Available from: http://dx.doi.org/10.1186/s12966-016-0452-0

9. Hunter S, Rosu A, Hesketh KD, Rhodes RE, Rinaldi CM, Rodgers W, et al. Objectively Measured Environmental Correlates of Toddlers’ Physical Activity and Sedentary Behavior. Pediatr Exerc Sci. 2019 Apr;1–8.

10. Nyunt MSZ, Shuvo FK, Eng JY, Yap KB, Scherer S, Hee LM, et al. Objective and subjective measures of neighborhood environment (NE): Relationships with transportation physical activity among older persons. Int J Behav Nutr Phys Act [Internet]. 2015;12(1):1–10. Available from: https://www.scopus.com/inward/record.uri?eid=2-s2.0-84941646860&doi=10.1186%2Fs12966-015-0276-3&partnerID=40&md5=7c002d006e739b1936961418db9c4343

11. McGowan EL, Fuller D, Cutumisu N, North S, Courneya KS. The role of the built environment in a randomized controlled trial to increase physical activity among men with prostate cancer: the PROMOTE trial. Support Care Cancer [Internet]. 2017;25(10):2993–6. Available from: https://www.scopus.com/inward/record.uri?eid=2-s2.0-85021298995&doi=10.1007%2Fs00520-017-3798-1&partnerID=40&md5=0d1e74f0db34d5898933e93bc2cbc59a

12. Li X, Santi P, Courtney TK, Verma SK, Ratti C. Investigating the association between streetscapes and human walking activities using Google Street View and human trajectory data. Trans GIS [Internet]. 2018;22(4):1029–44. Available from: https://www.scopus.com/inward/record.uri?eid=2-s2.0-85052506201&doi=10.1111%2Ftgis.12472&partnerID=40&md5=ef9ecfb755444ad476207218a91361d2

13. Hajna S, Ross NA, Joseph L, Harper S, Dasgupta K. Neighbourhood Walkability and Daily Steps in Adults with Type 2 Diabetes. PLoS One. 2016 Mar;11(3).

14. Brown SC, Pantin H, Lombard J, Toro M, Huang S, Plater-Zyberk E, et al. Walk Score® : Associations with Purposive Walking in Recent Cuban Immigrants. Am J Prev Med [Internet]. 2013;45(2):202–6. Available from: https://www.scopus.com/inward/record.uri?eid=2-s2.0-84880283726&doi=10.1016%2Fj.amepre.2013.03.021&partnerID=40&md5=28dc1625343faedce174d2f80851ffbc

15. Marquet O, Hipp AJ. Worksite built environment and objectively measured physical activity while at work: An analysis using perceived and objective Walkability and greenness. J Environ Health. 2019 Mar;81(7):20–6.

16. Frank LD, Sallis JF, Saelens BE, Leary L, Cain L, Conway TL, et al. The development of a walkability index: Application to the neighborhood quality of life study. Br J Sports Med. 2010;44(13):924–33.

17. Travers C, Dixon A, Laurence A, Niblett S, King K, Lewis P, et al. Retirement Health and Lifestyle Study: Australian Neighborhood Environments and Physical Activity in Older Adults. Environ Behav [Internet]. 2018;50(4):426–53. Available from: https://www.scopus.com/inward/record.uri?eid=2-s2.0-85044950409&doi=10.1177%2F0013916517707294&partnerID=40&md5=0436c04ba7977ffc7e6a86711fc8bdc1

18. Frank LD, Kerr J, Rosenberg D, King A. Healthy aging and where you live: Community design relationships with physical activity and body weight in older Americans. J Phys Act Heal [Internet]. 2010;7(SUPPL.1):S82–90. Available from: https://www.scopus.com/inward/record.uri?eid=2-s2.0-77749240194&partnerID=40&md5=a77f58e537c270a8b802e306c06847e1

19. Berke EM, Koepsell TD, Moudon A V, Hoskins RE, Larson EB. Association of the built environment with physical activity and obesity in older persons. Am J Public Health [Internet]. 2007;97(3):486–92. Available from: https://www.scopus.com/inward/record.uri?eid=2-s2.0-33847729863&doi=10.2105%2FAJPH.2006.085837&partnerID=40&md5=d3b67d7f5edc5916cb1ee6b2b3e4d5ae

20. Portegijs E, Keskinen KE, Eronen J, Saajanaho M. Older Adults ’ Physical Activity and the Relevance of Distances to Neighborhood Destinations and Barriers to Outdoor Mobility. Front public Heal. 2020;8: 335(August):1–11.

21. Marquet O, Hipp JA, Miralles-Guasch C. Neighborhood walkability and active ageing: A difference in differences assessment of active transportation over ten years. J Transp Heal [Internet]. 2017;7(September):190–201. Available from: http://linkinghub.elsevier.com/retrieve/pii/S221414051730097X

22. Portegijs E, Keskinen KE, Tsai L-T, Rantanen T, Rantakokko M. Physical Limitations, Walkability, Perceived Environmental Facilitators and Physical Activity of Older Adults in Finland. IInternational J Environ Res public Heal. 2017 Mar;14(3).

23. Cho G-H, Rodríguez DA. Neighborhood design, neighborhood location, and three types of walking: Results from the Washington DC area. Environ Plan B Plan Des [Internet]. 2015;42(3):526–40. Available from: https://www.scopus.com/inward/record.uri?eid=2-s2.0-84929334246&doi=10.1068%2Fb130222p&partnerID=40&md5=aaeb52bc8a09bfbdf229401de70ba2ae

24. Li Y, Yatsuya H, Hanibuchi T, Hirakawa Y, Ota A, Uemura M, et al. The association between objective measures of residence and worksite neighborhood environment, and self-reported leisure-time physical activities: The Aichi Workers’ Cohort Study. Prev Med Reports [Internet]. 2018;11:282–9. Available from: https://www.scopus.com/inward/record.uri?eid=2-s2.0-85050875582&doi=10.1016%2Fj.pmedr.2018.07.007&partnerID=40&md5=8b54fc0d8b4dd5b2e18618aff2524e99

25. Lovasi GS, Jacobson JS, Quinn JW, Neckerman KM, Ashby-Thompson MN, Rundle A. Is the environment near home and school associated with physical activity and adiposity of urban preschool children? J Urban Heal [Internet]. 2011;88(6):1143–57. Available from: https://www.scopus.com/inward/record.uri?eid=2-s2.0-84855256118&doi=10.1007%2Fs11524-011-9604-3&partnerID=40&md5=da0ac15c6a6a44c624367148783f6e67

26. McCormack GR, Shiell A, Doyle-Baker PK, Friedenreich CM, Sandalack BA. Subpopulation differences in the association between neighborhood urban form and neighborhood-based physical activity. Heal Place [Internet]. 2014;28:109–15. Available from: https://www.scopus.com/inward/record.uri?eid=2-s2.0-84899884707&doi=10.1016%2Fj.healthplace.2014.04.001&partnerID=40&md5=1e70ffe872a3c7ad08e75430bb319c9f

27. Rundle AG, Chen Y, Quinn JW, Rahai N, Bartley K, Mooney SJ, et al. Development of a Neighborhood Walkability Index for Studying Neighborhood Physical Activity Contexts in Communities across the U.S. over the Past Three Decades. J Urban Health. 2019 Jun;

28. Sugiyama T, Cole R, Koohsari MJ, Kynn M, Sallis JF, Owen N. Associations of local-area walkability with disparities in residents’ walking and car use. Prev Med (Baltim). 2019 Mar;120:126–30.

29. Tamura K, Wilson JS, Goldfeld K, Puett RC, Klenosky DB, Harper WA, et al. Accelerometer and GPS Data to Analyze Built Environments and Physical Activity. Res Q Exerc Sport [Internet]. 2019 Jun;90(3):395–402. Available from: https://doi.org/10.1080/02701367.2019.1609649

30. Eom HJ, Cho GH. Exploring thresholds of built environment characteristics for walkable communities: Empirical evidence from the Seoul Metropolitan area. Transp Res Part D Transp Environ [Internet]. 2015;40:76–86. Available from: http://dx.doi.org/10.1016/j.trd.2015.07.005

31. Gell NM, Rosenberg DE, Carlson J, Kerr J, Belza B. Built environment attributes related to GPS measured active trips in mid-life and older adults with mobility disabilities. Disabil Health J. 2015 Apr;8(2):290–5.

32. Grasser G, van Dyck D, Titze S, Stronegger WJ. A European perspective on GIS-based walkability and active modes of transport. Eur J Public Health. 2016 Feb;27(1):145–51.

33. Hajna S, Ross NA, Joseph L, Harper S, Dasgupta K. Neighbourhood walkability, daily steps and utilitarian walking in Canadian adults. BMJ Open. 2015;5(11):1–10.

34. Hajna S, Kestens Y, Daskalopoulou SS, Joseph L, Thierry B, Sherman M, et al. Neighbourhood walkability and home neighbourhood-based physical activity: an observational study of adults with type 2 diabetes. BMC Public Health. 2016;16.

35. Jack E, McCormack GR. The associations between objectively-determined and self-reported urban form characteristics and neighborhood-based walking in adults. Int J Behav Nutr Phys Act [Internet]. 2014;11(1). Available from: https://www.scopus.com/inward/record.uri?eid=2-s2.0-84902084844&doi=10.1186%2F1479-5868-11-71&partnerID=40&md5=37f5cf23430d3c60a305a2c2813f33de

36. James P, Hart JE, Hipp JA, Mitchell JA, Kerr J, Hurvitz PM, et al. GPS-Based Exposure to Greenness and Walkability and Accelerometry-Based Physical Activity. Cancer epodemiology biomarkers Prev. 2017;26(4):525–32.

37. Janssen I, King N. Walkable school neighborhoods are not playable neighborhoods. Heal Place. 2015;35:66–9.

38. Zandieh R, Flacke J, Martinez J, Jones P, van Maarseveen M. Do Inequalities in Neighborhood Walkability Drive Disparities in Older Adults’ Outdoor Walking? Int J Environ Res Public Health. 2017 Jul;14(7).

39. Carlson JA, Sallis JF, Saelens BE, Frank LD, Kerr J, Cain KL, et al. Interactions between Psychosocial and Built Environment Factors in Explaining Older Adults’ Physical Activity. Prev Med (Baltim) [Internet]. 2012;54(1):68–73. Available from: https://www.scopus.com/inward/record.uri?eid=2-s2.0-84855506359&doi=10.1016%2Fj.ypmed.2011.10.004&partnerID=40&md5=ee6711efac0b5f7302f44a3284f4ed07

40. Kikuchi H, Nakaya T, Hanibuchi T, Fukushima N, Amagasa S, Oka K, et al. Objectively Measured Neighborhood Walkability and Change in Physical Activity in Older Japanese Adults: A Five-Year Cohort Study. Int J Environ Res Public Health. 2018;15(9).

41. King AC, Sallis JF, Frank LD, Saelens BE, Cain K, Conway TL, et al. Aging in neighborhoods differing in walkability and income: Associations with physical activity and obesity in older adults. Soc Sci Med. 2011 Nov;73(10):1525–33.

42. Lotfi S, Koohsari MJ. Neighborhood Walkability in a City within a Developing Country. J Urban Plan Dev -ASCE [Internet]. 2011;137(4):402–8. Available from: https://www.scopus.com/inward/record.uri?eid=2-s2.0-84855929670&doi=10.1061%2F%28ASCE%29UP.1943-5444.0000085&partnerID=40&md5=eba4b17b3dfa21ff2415561de8739901

43. Todd M, Adams MA, Kurka J, Conway TL, Cain KL, Buman MP, et al. GIS-measured walkability, transit, and recreation environments in relation to older Adults’ physical activity: A latent profile analysis. Prev Med (Baltim) [Internet]. 2016 Dec;93(1):57–63. Available from: https://linkinghub.elsevier.com/retrieve/pii/S0091743516302778

44. Van Holle V, Van Cauwenberg J, Van Dyck D, Deforche B, Van de Weghe N, De Bourdeaudhuij I. Relationship between neighborhood walkability and older adults’ physical activity: results from the Belgian Environmental Physical Activity Study in Seniors (BEPAS Seniors). Int J Behav Nutr Phys Act. 2014;11(1):1–9.

45. Van Holle V, Van Cauwenberg J, Deforche B, Van de Weghe N, De Bourdeaudhuij I, Van Dyck D. Do psychosocial factors moderate the association between objective neighborhood walkability and older adults’ physical activity? Heal Place. 2015 Jul;34:118–25.

46. Van Holle V, Van Cauwenberg J, Gheysen F, Van Dyck D, Deforche B, Van De Weghe N, et al. The association between Belgian older adults’ physical functioning and physical activity: What is the moderating role of the physical environment? PLoS One [Internet]. 2016;11(2):1–17. Available from: https://www.scopus.com/inward/record.uri?eid=2-s2.0-84960532651&doi=10.1371%2Fjournal.pone.0148398&partnerID=40&md5=9d57944905c7328fed5dd7ab3e695ce8

47. Adams MA, Todd M, Kurka J, Conway TL, Cain KL, Frank LD, et al. Patterns of Walkability, Transit, and Recreation Environment for Physical Activity. Am J Prev Med. 2015;49(6):878–87.

48. Arvidsson D, Kawakami N, Ohlsson H, Sundquist K. Physical activity and concordance between objective and perceived walkability. Med Sci Sports Exerc. 2012;44(2):280–7.

49. Arvidsson D, Eriksson U, Lonn SL, Sundquist K. Neighborhood Walkability, Income, and Hour-by-Hour Physical Activity Patterns. Med Sci Sports Exerc. 2013;45(4):698–705.

50. Badland H, Mavoa S, Boulangé C, Eagleson S, Gunn L, Stewart J, et al. Identifying, creating, and testing urban planning measures for transport walking: Findings from the Australian national liveability study. J Transp Heal [Internet]. 2016;5:151–62. Available from: https://www.scopus.com/inward/record.uri?eid=2-s2.0-85011103517&doi=10.1016%2Fj.jth.2016.08.010&partnerID=40&md5=e2c3d93956ef5eeb2c675b5cead6b2cb

51. Cerin E, Frank LD, Sallis JF, Saelens BE, Conway TL, Chapman JE, et al. From neighborhood design and food options to residents’ weight status. Appetite [Internet]. 2011;56(3):693–703. Available from: https://www.scopus.com/inward/record.uri?eid=2-s2.0-79952984863&doi=10.1016%2Fj.appet.2011.02.006&partnerID=40&md5=566c7a89f09ec65bdf9f2f45e1f352c9

52. Christian HE, Bull FC, Middleton NJ, Knuiman MW, Divitini ML, Hooper P, et al. How important is the land use mix measure in understanding walking behaviour? Results from the RESIDE study. Int J Behav Nutr Phys Act [Internet]. 2011;8. Available from: https://www.scopus.com/inward/record.uri?eid=2-s2.0-79957861655&doi=10.1186%2F1479-5868-8-55&partnerID=40&md5=e81fc1ccc86e1050ddf215f8c7144d54

53. Christiansen LB, Madsen T, Schipperijn J, Ersboll AK, Troelsen J. Variations in active transport behavior among different neighborhoods and across adult lifestages. J Transp Heal [Internet]. 2014 Dec;1(4):316–25. Available from: https://www.scopus.com/inward/record.uri?eid=2-s2.0-84919332846&doi=10.1016%2Fj.jth.2014.10.002&partnerID=40&md5=c4c8ccc33c7528b4161907cef80eca62

54. Chum A, Atkinson P, O’Campo P. Does time spent in the residential neighbourhood moderate the relationship between neighbourhood walkability and transport-related walking? a cross-sectional study from Toronto, Canada. BMJ Open [Internet]. 2019;9(4). Available from: https://www.scopus.com/inward/record.uri?eid=2-s2.0-85063953906&doi=10.1136%2Fbmjopen-2018-023598&partnerID=40&md5=dea48f5d9fde4e0ba5fe0cc8fad269da

55. Cruise SM, Hunter RF, Kee F, Donnelly M, Ellis G, Tully MA. A comparison of road- and footpath-based walkability indices and their associations with active travel. J Transp Heal. 2017;6(July 2016):119–27.

56. Curl A, Kearns A, Macdonald L, Mason P, Ellaway A. Can walking habits be encouraged through area-based regeneration and relocation? A longitudinal study of deprived communities in Glasgow, UK. J Transp Heal [Internet]. 2018;10:44–55. Available from: https://www.scopus.com/inward/record.uri?eid=2-s2.0-85049337692&doi=10.1016%2Fj.jth.2018.06.004&partnerID=40&md5=d31b1d871a8e8a8a743ee0e965aaa899

57. D’Haese S, Van Dyck D, De Bourdeaudhuij I, Deforche B, Cardon G. The association between objective walkability, neighborhood socio-economic status, and physical activity in Belgian children. Int J Behav Nutr Phys Act. 2014;11(1):7–14.

58. De Meester F, Van Dyck D, De Bourdeaudhuij I, Deforche B, Sallis JF, Cardon G. Active living neighborhoods: Is neighborhood walkability a key element for Belgian adolescents? BMC Public Health [Internet]. 2012;12(1):7. Available from: http://www.biomedcentral.com/1471-2458/12/7

59. Dygryn J, Mitas J, Stelzer J. The Influence of Built Environment on Walkability Using Geographic Information System. J Hum Kinet. 2010 Jun;24:93–9.

60. Eriksson U, Arvidsson D, Gebel K, Ohlsson H, Sundquist K. Walkability parameters, active transportation and objective physical activity: moderating and mediating effects of motor vehicle ownership in a cross-sectional study. Int J Behav Nutr Phys Act. 2012 Oct;9.

61. Frank LD, Schmid TL, Sallis JF, Chapman J, Saelens BE. Linking objectively measured physical activity with objectively measured urban form: Findings from SMARTRAQ. Am J Prev Med. 2005 Feb;28(2 SUPPL. 2):117–25.

62. Frank LD, Saelens BE, Powell KE, Chapman JE. Stepping towards causation: Do built environments or neighborhood and travel preferences explain physical activity, driving, and obesity? Soc Sci Med. 2007;65(9):1898–914.

63. Frank LD, Kershaw SE, Chapman JE, Campbell M, Swinkels HM. The unmet demand for walkability: Disparities between preferences and actual choices for residential environments in Toronto and Vancouver. Can J Public Heal - Rev Can sante publique. 2015 Jul;106(1, 1):ES12–20.

64. Graziose MM, Gray HL, Quinn J, Rundle AG, Contento IR, Koch PA. Association Between the Built Environment in School Neighborhoods With Physical Activity Among New York City Children. Prev Chronic Dis [Internet]. 2016;13(110):1–11. Available from: https://www.scopus.com/inward/record.uri?eid=2-s2.0-84991492496&doi=10.5888%2Fpcd13.150581&partnerID=40&md5=8caedf3fb0627a70b493463466e8e219

65. Hinckson E, Cerin E, Mavoa S, Smith M, Badland H, Stewart T, et al. Associations of the perceived and objective neighborhood environment with physical activity and sedentary time in New Zealand adolescents. Int J Behav Nutr Phys Act [Internet]. 2017;14(1):145. Available from: https://www.scopus.com/inward/record.uri?eid=2-s2.0-85043360385&doi=10.1186%2Fs12966-017-0597-5&partnerID=40&md5=6a17527e93e54cb71e470f5d45df77fd

66. Huang R, Moudon A V, Zhou C, Saelens BE. Higher residential and employment densities are associated with more objectively measured walking in the home neighborhood. J Transp Heal [Internet]. 2019;12:142–51. Available from: https://www.scopus.com/inward/record.uri?eid=2-s2.0-85060235448&doi=10.1016%2Fj.jth.2018.12.002&partnerID=40&md5=b5f926e3dea4c491f374122e90eaa92e

67. Kelly C, Lian M, Struthers J, Kammrath A. Walking to Work: The Roles of Neighborhood Walkability and Socioeconomic Deprivation. J Phys Act Heal. 2015 Jun;12(1):S70–5.

68. Kerr J, Norman GJ, Adams MA, Ryan S, Frank LD, Sallis JF, et al. Do neighborhood environments moderate the effect of physical activity lifestyle interventions in adults? Heal Place. 2010 Sep;16(5):1–7.

69. Kerr J, Norman G, Millstein R, Adams MA, Morgan C, Langer RD, et al. Neighborhood environment and physical activity among older women: Findings from the San Diego cohort of the women’s health initiative. J Phys Act Heal [Internet]. 2014;11(6):1070–7. Available from: https://www.scopus.com/inward/record.uri?eid=2-s2.0-84910006911&doi=10.1123%2Fjpah.2012-0159&partnerID=40&md5=b3e75e53de1e7538a874b4f797a61dcb

70. Kligerman M, Sallis JF, Ryan S, Frank LD, Nader PR. Association of neighborhood design and recreation environment variables with physical activity and body mass index in adolescents. Am J Heal Promot [Internet]. 2007;21(4):274–7. Available from: https://www.scopus.com/inward/record.uri?eid=2-s2.0-33947205048&doi=10.4278%2F0890-1171-21.4.274&partnerID=40&md5=84fc91bb3a78ea5d52439228d97ceed6

71. Koohsari MJ, Owen N, Cerin E, Giles-Corti B, Sugiyama T. Walkability and walking for transport: characterizing the built environment using space syntax. Int J Behav Nutr Phys Act. 2016 Nov;13.

72. Learnihan V, Van Niel KP, Giles-Corti B, Knuiman M. Effect of Scale on the Links between Walking and Urban Design. Geogr Res [Internet]. 2011;49(2):183–91. Available from: https://www.scopus.com/inward/record.uri?eid=2-s2.0-79955521869&doi=10.1111%2Fj.1745-5871.2011.00689.x&partnerID=40&md5=b529604a6563b7553ec5d545677da175

73. Maddison R, Hoorn S V, Jiang Y, Mhurchu CN, Exeter D, Dorey E, et al. The environment and physical activity: The influence of psychosocial, perceived and built environmental factors. Int J Behav Nutr Phys Act [Internet]. 2009;6. Available from: https://www.scopus.com/inward/record.uri?eid=2-s2.0-66149156287&doi=10.1186%2F1479-5868-6-19&partnerID=40&md5=30ece99f4521edd5a1e36c7c2e1123b5

74. McCormack GR, Shiell A, Giles-Corti B, Begg S, Veerman JL, Geelhoed E, et al. The association between sidewalk length and walking for different purposes in established neighborhoods. Int J Behav Nutr Phys Act [Internet]. 2012;9. Available from: https://www.scopus.com/inward/record.uri?eid=2-s2.0-84864405616&doi=10.1186%2F1479-5868-9-92&partnerID=40&md5=76c711b394310953e7b10f8044ae3fbe

75. McGrath LJ, Hinckson EA, Hopkins WG, Mavoa S, Witten K, Schofield G. Associations Between the Neighborhood Environment and Moderate-to-Vigorous Walking in New Zealand Children: Findings from the URBAN Study. Sport Med [Internet]. 2016;46(7):1003–17. Available from: https://www.scopus.com/inward/record.uri?eid=2-s2.0-84964335433&doi=10.1007%2Fs40279-016-0533-x&partnerID=40&md5=cd90742b34242d7a52159a95e33df5a1

76. Molina-García J, Queralt A. Neighborhood Built Environment and Socioeconomic Status in Relation to Active Commuting to School in Children. J Phys Act Health [Internet]. 2017 Oct;14(10):761–5. Available from: https://www.scopus.com/inward/record.uri?eid=2-s2.0-85028998736&doi=10.1123%2Fjpah.2017-0033&partnerID=40&md5=099bb187734dd8e2fa221d6d0d61e8c7

77. Molina-Garcia J, Queralt A, Adams MA, Conway TL, Sallis JF. Neighborhood built environment and socioeconomic status in relation to multiple health outcomes in adolescents. Prev Med (Baltim) [Internet]. 2017 Dec;105:88–94. Available from: https://www.scopus.com/inward/record.uri?eid=2-s2.0-85028953352&doi=10.1016%2Fj.ypmed.2017.08.026&partnerID=40&md5=d08570d3dddd0f352faca0291c68ae3e

78. Norman GJ, Carlson JA, O’Mara S, Sallis JF, Patrick K, Frank LD, et al. Neighborhood preference, walkability and walking in overweight/obese men. Am J Health Behav. 2013;37(2):277–82.

79. Oliver M, Mavoa S, Badland H, Parker K, Donovan P, Kearns RA, et al. Associations between the neighbourhood built environment and out of school physical activity and active travel: An examination from the Kids in the City study. Heal Place [Internet]. 2015;36:57–64. Available from: https://www.scopus.com/inward/record.uri?eid=2-s2.0-84942769038&doi=10.1016%2Fj.healthplace.2015.09.005&partnerID=40&md5=c8885b7e8b876049c21b9b667499a438

80. Oluyomi AO, Whitehead LW, Burau KD, Symanski E, Kohl HW, Bondy M. Physical activity guideline in Mexican-Americans: Does the built environment play a role? J Immigr Minor Heal [Internet]. 2014;16(2):244–55. Available from: https://www.scopus.com/inward/record.uri?eid=2-s2.0-84896392799&doi=10.1007%2Fs10903-012-9724-1&partnerID=40&md5=7c3a60bfc3490eedcaa03c023febc63a

81. Owen N, Cerin E, Leslie E, duToit L, Coffee N, Frank LD, et al. Neighborhood walkability and the walking behavior of Australian adults. Am J Prev Med. 2007 Nov;33(5):387–95.

82. Perez LG, Conway TL, Arredondo EM, Elder JP, Kerr J, McKenzie TL, et al. Where and when adolescents are physically active: Neighborhood environment and psychosocial correlates and their interactions. Prev Med (Baltim) [Internet]. 2017;105:337–44. Available from: https://www.scopus.com/inward/record.uri?eid=2-s2.0-85030850491&doi=10.1016%2Fj.ypmed.2017.10.010&partnerID=40&md5=8fcb96d40f4058ed5568d3d8b9df09e0

83. Ribeiro AI, Hoffimann E. Development of a neighbourhood walkability index for porto metropolitan area. How strongly is walkability associated with walking for transport? Int J Environ Res Public Health. 2018;15(12).

84. Riley DL, Mark AE, Kristjansson E, Sawada MC, Reid RD. Neighbourhood walkability and physical activity among family members of people with heart disease who participated in a randomized controlled trial of a behavioural risk reduction intervention. Heal Place. 2013;21:148–55.

85. Rundle AG, Sheehan DM, Quinn JW, Bartley K, Eisenhower D, Bader MMD, et al. Using GPS Data to Study Neighborhood Walkability and Physical Activity. Am J Prev Med. 2016 Mar;50(3):E65–72.

86. Sallis JF, Saelens BE, Frank LD, Conway TL, Slymen DJ, Cain KL, et al. Neighborhood Built Environment and Income: Examining Multiple Health Outcomes. Soc Sci Med [Internet]. 2009;68(7):1285–93. Available from: https://www.scopus.com/inward/record.uri?eid=2-s2.0-62249147570&doi=10.1016%2Fj.socscimed.2009.01.017&partnerID=40&md5=35f2de2afdb47e703142557e17b28029

87. Salvo D, Reis RS, Stein AD, Rivera J, Martorell R, Pratt M. Characteristics of the built environment in relation to objectively measured physical activity among Mexican adults, 2011. Prev Chronic Dis [Internet]. 2014;11. Available from: https://www.scopus.com/inward/record.uri?eid=2-s2.0-84916638294&doi=10.5888%2Fpcd11.140047&partnerID=40&md5=f6e719f8858a26cb30a8c6feae88acce

88. Shimura H, Sugiyama T, Winkler E, Owen N. High Neighborhood Walkability Mitigates Declines in Middle-to-Older Aged Adults’ Walking for Transport. J Phys Act Heal. 2012;9(7):1004–8.

89. Shimura H, Winkler E, Owen N. Individual, psychosocial, and environmental correlates of 4-year declines in walking among middle-to-older aged adults. J Phys Act Heal [Internet]. 2014;11(6):1078–84. Available from: https://www.scopus.com/inward/record.uri?eid=2-s2.0-84910006471&doi=10.1123%2Fjpah.2012-0244&partnerID=40&md5=d7d4cfc10fcd5c9864b42f3dcdbedc25

90. Siqueira Reis R, Hino AAF, Rech CR, Kerr J, Hallal PC. Walkability and physical activity: Findings from curitiba, brazil. Am J Prev Med [Internet]. 2013;45(3):269–75. Available from: https://www.scopus.com/inward/record.uri?eid=2-s2.0-84883025841&doi=10.1016%2Fj.amepre.2013.04.020&partnerID=40&md5=f1f73c3233c59235f1d233ce645c8b9c

91. Smith L, Panter J, Ogilvie D. Characteristics of the environment and physical activity in midlife: Findings from UK Biobank. Prev Med (Baltim) [Internet]. 2019;118:150–8. Available from: https://www.scopus.com/inward/record.uri?eid=2-s2.0-85055677100&doi=10.1016%2Fj.ypmed.2018.10.024&partnerID=40&md5=91bf3170f66ada98ad72e562356c9bae

92. Sugiyama T, Howard NJ, Paquet C, Coffee NT, Taylor AW, Daniel M. Do Relationships Between Environmental Attributes and Recreational Walking Vary According to Area-Level Socioeconomic Status? J Urban Heal [Internet]. 2015;92(2):253–64. Available from: https://www.scopus.com/inward/record.uri?eid=2-s2.0-84939939245&doi=10.1007%2Fs11524-014-9932-1&partnerID=40&md5=08f8dc07fcfddd8d273cdcc162a9c8b3

93. Sundquist K, Eriksson U, Kawakami N, Skog L, Ohlsson H, Arvidsson D. Neighborhood walkability, physical activity, and walking behavior: The Swedish Neighborhood and Physical Activity (SNAP) study. Soc Sci Med. 2011;72(8):1266–73.

94. Van Dyck D, Cerin E, Cardon G, Deforche B, Sallis JF, Owen N, et al. Physical activity as a mediator of the associations between neighborhood walkability and adiposity in Belgian adults. Heal Place [Internet]. 2010;16(5):952–60. Available from: http://dx.doi.org/10.1016/j.healthplace.2010.05.011

95. Van Dyck D, Cardon G, Deforche B, Sallis JF, Owen N, De Bourdeaudhuij I. Neighborhood SES and walkability are related to physical activity behavior in Belgian adults. Prev Med (Baltim). 2010;50(SUPPL.):74–9.

96. Van Dyck D, Cardon G, Deforche B, De Bourdeaudhuij I, Van Dyck D, Cardon G, et al. Do adults like living in high-walkable neighborhoods? Associations of walkability parameters with neighborhood satisfaction and possible mediators. Heal Place [Internet]. 2011 Jul;17(4):971–7. Available from: https://www.scopus.com/inward/record.uri?eid=2-s2.0-79958072347&doi=10.1016%2Fj.healthplace.2011.04.001&partnerID=40&md5=537e6c03af476718cdd4dc4bb7813abd

97. Villanueva K, Knuiman M, Nathan A, Giles-Corti B, Christian H, Foster S, et al. The impact of neighborhood walkability on walking: Does it differ across adult life stage and does neighborhood buffer size matter? Heal Place [Internet]. 2014;25:43–6. Available from: http://dx.doi.org/10.1016/j.healthplace.2013.10.005

98. Wei YD, Xiao W, Wen M, Wei R. Walkability, Land Use and Physical Activity. Sustainability. 2016;8(1).

99. Witten K, Blakely T, Bagheri N, Badland H, Ivory V, Pearce J, et al. Neighborhood built environment and transport and leisure physical activity: Findings using objective exposure and outcome measures in New Zealand. Environ Health Perspect [Internet]. 2012;120(7):971–7. Available from: https://www.scopus.com/inward/record.uri?eid=2-s2.0-84864121850&doi=10.1289%2Fehp.1104584&partnerID=40&md5=2e3cd9eb4fb40f9b2c02bb00709f38f7

100. Yi LY, Samat N, Wan Muda WM. Accelerometer-measured physical activity and its relationship with Body Mass Index (BMI) and Waist Circumference (WC) measurements: A cross-sectional study on Malaysian adults. Malays J Nutr [Internet]. 2017;23(3):397–408. Available from: https://www.scopus.com/inward/record.uri?eid=2-s2.0-85040450954&partnerID=40&md5=1b7d7b6d9f0d50aaadfdeb13eebb62fb

101. Bodeker M. Walking and Walkability in Pre-Set and Self-Defined Neighborhoods: A Mental Mapping Study in Older Adults. Int J Environ Res Public Health [Internet]. 2018 Jul;15(7). Available from: https://www.scopus.com/inward/record.uri?eid=2-s2.0-85049466191&doi=10.3390%2Fijerph15071363&partnerID=40&md5=a6c4dd4fd88153d1b62a2db742895819

102. Reyer M, Fina S, Siedentop S, Schlicht W. Walkability is Only Part of the Story: Walking for Transportation in Stuttgart, Germany. Int J Environ Res Public Health. 2014 Jun;11(6):5849–65.

103. Mayne DJ, Morgan GG, Willmore A, Rose N, Jalaludin B, Bambrick H, et al. An objective index of walkability for research and planning in the Sydney Metropolitan Region of New South Wales, Australia: an ecological study. Int J Health Geogr. 2013;12.

104. Mayne DJ, Morgan GG, Jalaludin BB, Bauman AE. The contribution of area-level walkability to geographic variation in physical activity: a spatial analysis of 95,837 participants from the 45 and Up Study living in Sydney, Australia. Popul Health Metr. 2017 Oct;15.

105. Shay E, Khattak AJ. Household travel decision chains: Residential environment, automobile ownership, trips and mode choice. Int J Sustain Transp [Internet]. 2012;6(2):88–110. Available from: https://www.scopus.com/inward/record.uri?eid=2-s2.0-80052546206&doi=10.1080%2F15568318.2011.560363&partnerID=40&md5=58857cd3888202975072cbd057469a7f

106. Lee H, Kang H-M, Ko Y-J, Kim H-S, Kim Y-J, Bae WK, et al. Influence of urban neighbourhood environment on physical activity and obesity-related diseases. Public Health. 2015 Sep;129(9):1204–10.

107. Liao Y, Lin C-Y, Lai T-F, Chen Y-J, Kim B, Park J-H. Walk score® and its associations with older adults’ health behaviors and outcomes. Int J Environ Res Public Health [Internet]. 2019;16(4). Available from: https://www.scopus.com/inward/record.uri?eid=2-s2.0-85061972589&doi=10.3390%2Fijerph16040622&partnerID=40&md5=f9b36cb969d6e8547b340cd12aa1324f

108. Takahashi PY, Baker MA, Cha S, Targonski P V. A cross-sectional survey of the relationship between walking, biking, and the built environment for adults aged over 70 years. Risk Manag Healthc Policy [Internet]. 2012;5:35–41. Available from: https://www.scopus.com/inward/record.uri?eid=2-s2.0-84864493229&partnerID=40&md5=ab50807ea61d29162278b3ada94491d2

109. Barnes R, Winters M, Ste-Marie N, McKay H, Ashe MC. Age and retirement status differences in associations between the built environment and active travel behaviour. J Transp Heal [Internet]. 2016;3(4):513–22. Available from: https://www.scopus.com/inward/record.uri?eid=2-s2.0-84962144853&doi=10.1016%2Fj.jth.2016.03.003&partnerID=40&md5=d29dd67ed5d8a19984cbc97182dc3514

110. Boisjoly G, Wasfi R, El-Geneidy A. How much is enough? Assessing the influence of neighborhood walkability on undertaking 10-minute walks. J Transp Land Use. 2018;11(1):143–51.

111. Cole R, Dunn P, Hunter I, Owen N, Sugiyama T. Walk Score and Australian adults’ home-based walking for transport. Heal Place [Internet]. 2015;35:60–5. Available from: https://www.scopus.com/inward/record.uri?eid=2-s2.0-84939201361&doi=10.1016%2Fj.healthplace.2015.06.011&partnerID=40&md5=8f27078ae4b84a49aed709966edb9db9

112. Duncan DT, Méline J, Kestens Y, Day K, Elbel B, Trasande L, et al. Walk score, transportation mode choice, and walking among french adults: A GPS, accelerometer, and mobility survey study. Int J Environ Res Public Health [Internet]. 2016;13(6):1–14. Available from: https://www.scopus.com/inward/record.uri?eid=2-s2.0-84975291140&doi=10.3390%2Fijerph13060611&partnerID=40&md5=2a8c65b914893d518eacee500671547c

113. Forjuoh SN, Ory MG, Won J, Towne SD, Wang S, Lee C. Determinants of Walking among Middle-Aged and Older Overweight and Obese Adults: Sociodemographic, Health, and Built Environmental Factors. J Obes [Internet]. 2017;2017. Available from: https://www.scopus.com/inward/record.uri?eid=2-s2.0-85024498461&doi=10.1155%2F2017%2F9565430&partnerID=40&md5=4e7c5f3afa812434b62b48c3c9ab05bb

114. Han M, Ye X, Preciado P, Williams S, Campos I, Bonner M, et al. Relationships between Neighborhood Walkability and Objectively Measured Physical Activity Levels in Hemodialysis Patients. Blood Purif. 2018;45(1–3):236–44.

115. Hirsch JA, Moore KA, Evenson KR, Rodriguez DA, Roux AVD. Walk Score® and Transit Score® and Walking in the Multi-Ethnic Study of Atherosclerosis. Am J Prev Med [Internet]. 2013;45(2):158–66. Available from: https://www.scopus.com/inward/record.uri?eid=2-s2.0-84880323998&doi=10.1016%2Fj.amepre.2013.03.018&partnerID=40&md5=29ec3f2a8242aebf3bdd7675f8192f45

116. Hirsch JA, Roux AVD, Moore KA, Evenson KR, Rodriguez DA. Change in walking and body mass index following residential relocation: The multi-ethnic study of atherosclerosis. Am J Public Health [Internet]. 2014;104(3):e49–56. Available from: https://www.scopus.com/inward/record.uri?eid=2-s2.0-84894092502&doi=10.2105%2FAJPH.2013.301773&partnerID=40&md5=4220c38ff73121985aab3b1bb9b3042a

117. Hwang L-D, Hurvitz PM, Duncan GE. Cross Sectional Association between Spatially Measured Walking Bouts and Neighborhood Walkability. Int J Environ Res Public Health. 2016;13(4).

118. Kelley EA, Kandula NR, Kanaya AM, Yen IH. Neighborhood walkability and walking for transport among South Asians in the masala study. J Phys Act Heal. 2016;13(5):514–9.

119. Lo BK, Graham ML, Folta SC, Paul LC, Strogatz D, Nelson ME, et al. Examining the associations betweenwalk score, perceived built environment, and physical activity behaviors among women participating in a community-randomized lifestyle change intervention trial: Strong hearts, healthy communities. Int J Environ Res Public Health [Internet]. 2019 Mar;16(5). Available from: https://www.scopus.com/inward/record.uri?eid=2-s2.0-85062817386&doi=10.3390%2Fijerph16050849&partnerID=40&md5=ea334b6f5e7133b06069b81bfaa658bb

120. McCormack GR, McLaren L, Salvo G, Blackstaffe A. Changes in Objectively-Determined Walkability and Physical Activity in Adults: A Quasi-Longitudinal Residential Relocation Study. Int J Environ Res Public Health. 2017;14(5).

121. Méline J, Chaix B, Pannier B, Ogedegbe G, Trasande L, Athens J, et al. Neighborhood walk score and selected Cardiometabolic factors in the French RECORD cohort study. BMC Public Health [Internet]. 2017 Dec;17(1):960. Available from: https://www.scopus.com/inward/record.uri?eid=2-s2.0-85038427730&doi=10.1186%2Fs12889-017-4962-8&partnerID=40&md5=d32f02cd7d6c7c39618a5661564fee80

122. Reid RER, Carver TE, Reid TGR, Picard-Turcot M-AM-A, Andersen KM, Christou N V, et al. Erratum to: Effects of Neighborhood Walkability on Physical Activity and Sedentary Behavior Long-Term Post-Bariatric Surgery. Obes Surg [Internet]. 2017 Jun;27(6):1595. Available from: https://www.scopus.com/inward/record.uri?eid=2-s2.0-85006467491&doi=10.1007%2Fs11695-016-2512-6&partnerID=40&md5=8842e01c86e017f9251644854e6d03a7

123. Salvo G, Lashewicz BM, Doyle-Baker PK, McCormack GR. A mixed methods study on the barriers and facilitators of physical activity associated with residential relocation. J Environ Public Health [Internet]. 2018;2018. Available from: https://www.scopus.com/inward/record.uri?eid=2-s2.0-85062414054&doi=10.1155%2F2018%2F1094812&partnerID=40&md5=68c07c2b79c023a82364468ea4f45ac5

124. Ross SET, Clennin MN, Dowda M, Colabianchi N, Pate RR, Taverno Ross SE, et al. Stepping It Up: Walking Behaviors in Children Transitioning from 5th to 7th Grade. Int J Environ Res Public Health [Internet]. 2018 Feb;15(2). Available from: https://www.scopus.com/inward/record.uri?eid=2-s2.0-85041480364&doi=10.3390%2Fijerph15020262&partnerID=40&md5=28bea1895347d6b8e4ca6e05cb046b8a

125. Towne SD, Won J, Lee S, Ory MG, Forjuoh SN, Wang S, et al. Using Walk Score^TM^ and Neighborhood Perceptions to Assess Walking Among Middle-Aged and Older Adults. J Community Health [Internet]. 2016 Oct;41(5):977–88. Available from: https://www.scopus.com/inward/record.uri?eid=2-s2.0-84961208535&doi=10.1007%2Fs10900-016-0180-z&partnerID=40&md5=986da26b89a48cb2164f8868ff84c0db

126. Towne SD, Lopez ML, Li Y, Smith ML, Warren JL, Evans AE, et al. Examining the Role of Income Inequality and Neighborhood Walkability on Obesity and Physical Activity among Low-Income Hispanic Adults. J Immigr Minor Heal [Internet]. 2018 Aug;20(4):854–64. Available from: https://www.scopus.com/inward/record.uri?eid=2-s2.0-85025458124&doi=10.1007%2Fs10903-017-0625-1&partnerID=40&md5=2e62667620326324883d02f558af16fb

127. Tuckel P, Milczarski W, Peter Tuckel, PhD; William Milczarski P. Walk Score(TM), Perceived Neighborhood Walkability, and Walking in the US. Am J Health Behav. 2015 Mar;39(2):242–56.

128. Twardzik E, Judd S, Bennett A, Hooker S, Howard V, Hutto B, et al. Walk Score and objectively measured physical activity within a national cohort. J Epidemiol Community Health [Internet]. 2019;73(6):549–56. Available from: https://www.scopus.com/inward/record.uri?eid=2-s2.0-85063937766&doi=10.1136%2Fjech-2017-210245&partnerID=40&md5=4d44334696301354590dd108f6a3443e

129. Wasfi RA, Dasgupta K, Eluru N, Ross NA. Exposure to walkable neighbourhoods in urban areas increases utilitarian walking: Longitudinal study of Canadians. J Transp Heal. 2015;3(4):440–7.

130. Wasfi R, Steinmetz-Wood M, Kestens Y. Place matters: A longitudinal analysis measuring the association between neighbourhood walkability and walking by age group and population center size in Canada. PLoS One. 2017;12(12).

131. Winters M, Barnes R, Venners S, Ste-Marie N, McKay H, Sims-Gould J, et al. Older adults’ outdoor walking and the built environment: Does income matter? Environmental health. BMC Public Health [Internet]. 2015 Sep;15(1):876. Available from: http://dx.doi.org/10.1186/s12889-015-2224-1

132. Chudyk AM, McKay HA, Winters M, Sims-Gould J, Ashe MC. Neighborhood walkability, physical activity, and walking for transportation: A cross-sectional study of older adults living on low income. BMC Geriatr. 2017;17(1):1–14.

133. Clarke P, Hirsch JA, Melendez R, Winters M, Gould JS, Ashe M, et al. Snow and Rain Modify Neighbourhood Walkability for Older Adults. Can J Aging. 2017 Jun;36(2):159–69.

134. Chiu M, Shah BR, Maclagan LC, Rezai M-R, Austin PC, Tu J V. Walk score® and the prevalence of utilitarian walking and obesity among Ontario adults: A cross-sectional study. Heal Reports [Internet]. 2015;26(7):3–10. Available from: https://www.scopus.com/inward/record.uri?eid=2-s2.0-84937691427&partnerID=40&md5=0308900f95834c7d4f49581bf4f16adf

135. Hirsch JA, Winters M, Clarke PJ, Ste-Marie N, McKay HA. The influence of walkability on broader mobility for Canadian middle aged and older adults: An examination of Walk Score^TM^ and the Mobility Over Varied Environments Scale (MOVES). Prev Med (Baltim). 2017;95:S60–7.

136. Thielman J, Manson H, Chiu M, Copes R, Rosella LC. Residents of highly walkable neighbourhoods in Canadian urban areas do substantially more physical activity: a cross-sectional analysis. C Open. 2016;4(4):E720–8.

137. Yang Y, Diez-Roux A V. Adults’ Daily Walking for Travel and Leisure: Interaction between Attitude Toward Walking and the Neighborhood Environment. Am J Heal Promot [Internet]. 2017;31(5):435–43. Available from: https://www.scopus.com/inward/record.uri?eid=2-s2.0-85028347122&doi=10.1177%2F0890117116669278&partnerID=40&md5=b96029ef43494f7edea580a295421fc5

138. Jensen WA, Brown BB, Smith KR, Brewer SC, Amburgey JW, McIff B. Active Transportation on a Complete Street: Perceived and Audited Walkability Correlates. Int J Environ Res Public Health. 2017;14(9).

139. Dills JE, Rutt CD, Mumford KG. Objectively Measuring Route-To-Park Walkability in Atlanta, Georgia. Environ Behav. 2012 Nov;44(6):841–60.

140. Richardson AS, Troxel WM, Ghosh-Dastidar MB, Beckman R, Hunter GP, DeSantis AS, et al. One size doesn’t fit all: cross-sectional associations between neighborhood walkability, crime and physical activity depends on age and sex of residents. BMC Public Health. 2017;17.

141. Strath SJ, Greenwald MJ, Isaacs R, Hart TL, Lenz EK, Dondzila CJ, et al. Measured and perceived environmental characteristics are related to accelerometer defined physical activity in older adults. Int J Behav Nutr Phys Act. 2012 Apr;9:40.

142. Michael YL, Gold R, Perrin NA, Hillier TA. Built environment and lower extremity physical performance: Prospective findings from the study of osteoporotic fractures in women. J Aging Health [Internet]. 2011;23(8):1246–62. Available from: https://www.scopus.com/inward/record.uri?eid=2-s2.0-81755181122&doi=10.1177%2F0898264311412597&partnerID=40&md5=3dca5d06ee81f500bc24d8bd13baa7ca

143. Doyle S, Kelly-Schwartz A, Schlossberg M, Stockard J. Active community environments and health: The relationship of walkable and safe communities to individual health. J Am Plan Assoc. 2006;72(1):19–31.

144. McCormack GR, Giles-Corti B, Timperio A, Wood G, Villanueva K. A cross-sectional study of the individual, social, and built environmental correlates of pedometer-based physical activity among elementary school children. Int J Behav Nutr Phys Act. 2011 Apr;8:30.

145. Michael YL, Carlson NE. Analysis of individual social-ecological mediators and moderators and their ability to explain effect of a randomized neighborhood walking intervention. Int J Behav Nutr Phys Act [Internet]. 2009;6. Available from: https://www.scopus.com/inward/record.uri?eid=2-s2.0-69249117823&doi=10.1186%2F1479-5868-6-49&partnerID=40&md5=484f870f40e9773d5a5562111790c9a0

146. Carter P, Bodicoat DH, Jones A, Khunti K, Davies MJ, Edwardson CL, et al. The impact of neighbourhood walkability on the effectiveness of a structured education programme to increase objectively measured walking. J Public Health (Bangkok). 2017 Mar;40(1):82–9.

147. Hosler AS, Gallant MP, Riley-Jacome M, Rajulu DT. Relationship between Objectively Measured Walkability and Exercise Walking among Adults with Diabetes. J Environ Public Health. 2014;
